# Fluctuation-driven mass-selective transport in dynamic nanopores

**DOI:** 10.1038/s41467-026-75540-5

**Published:** 2026-07-15

**Authors:** Zhiye Tang, Ken-ichi Otake, Hirotoshi Sakamoto, Susumu Kitagawa, Shinji Saito

**Affiliations:** 1 Institute for Molecular Science, Myodaiji, Okazaki, Japan; 2 The Graduate University for Advanced Studies (SOKENDAI), Myodaiji, Okazaki, Japan; 3 Institute for Integrated Cell-Material Sciences, Institute for Advanced Study, Kyoto University (KUIAS-iCeMS), Kyoto, Japan

**Keywords:** Molecular dynamics, Statistical mechanics

## Abstract

Precise molecular separation is essential in nanotechnology. However, static designs based on pore size and host-guest interactions often fail for species with nearly identical properties. To overcome this, we elucidate how nanopore dynamics govern transport and separation by establishing a general, predictive framework. We demonstrate that diffusion in a fluctuating periodic potential is maximized at an optimal fluctuation rate. Critically, this rate depends on molecular mass, translating subtle mass differences into significant kinetic disparities. By integrating this framework with quantum-chemical energy landscapes for archetypal soft porous crystals, we identify two factors governing selectivity: the magnitude of energy-barrier fluctuations and the alignment between nanopore dynamics and the energy-fluctuation rate that maximizes selectivity. These results establish nanopore dynamics as a key design dimension for controlling separation by tuning structural dynamics via ligand substitution or external fields, providing a theoretical foundation for the rational design of separation materials.

## Abstract (web summary)

The authors reveal how dynamic nanopore fluctuations amplify subtle mass differences into selective transport. Resonant activation at an optimal fluctuation rate enables separation beyond the mass-scaling limit, opening routes for materials design.

## Introduction

**Fig. 1:**
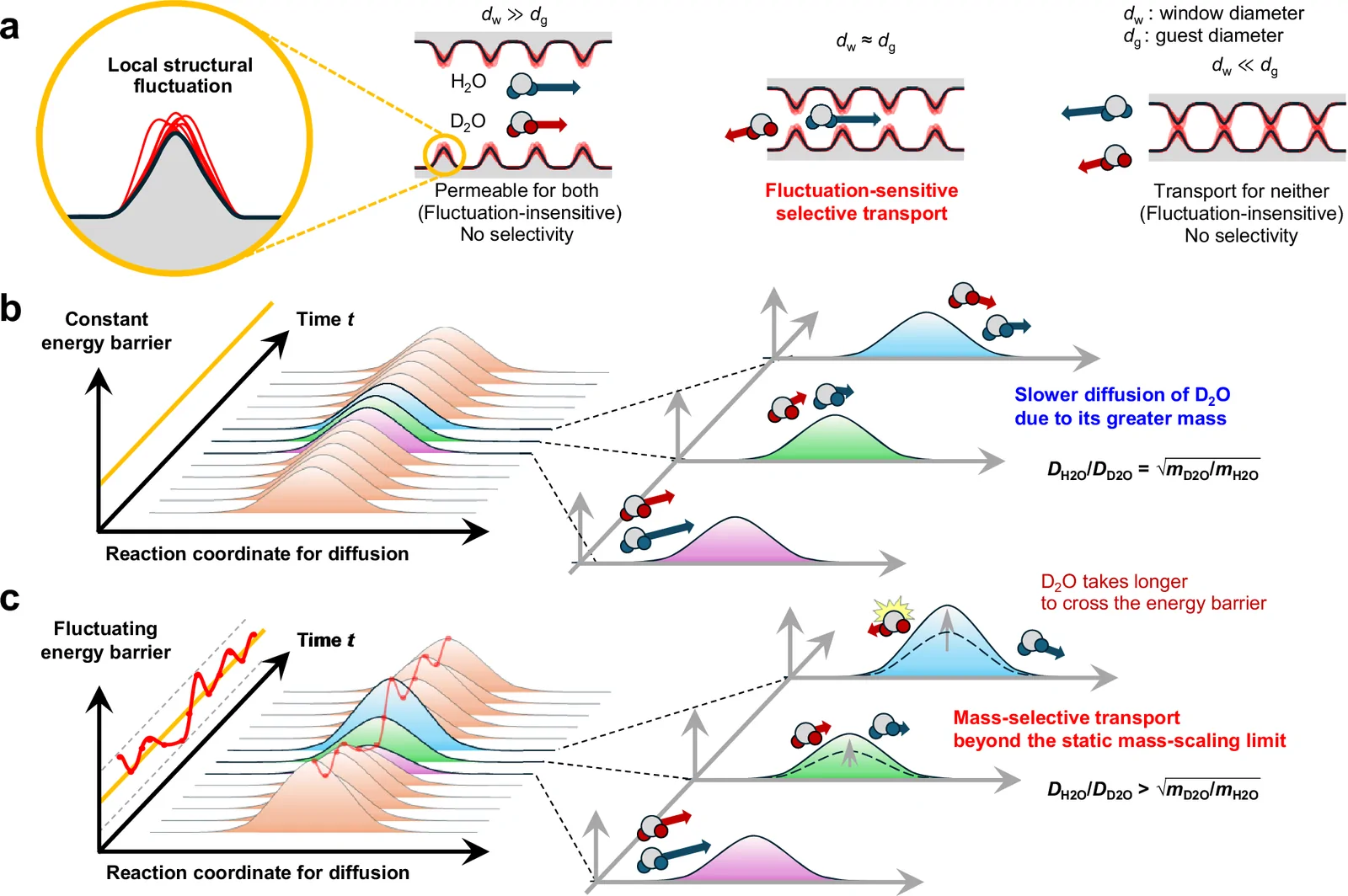
Fluctuation-driven mass-selective transport in dynamic nanopores. **a** Conceptual classification of transport regimes for two species with nearly identical properties (e.g., water isotopologues) across fluctuating energy barriers, depending on the relationship between the pore window diameter (*d*_w_) and guest diameter (*d*_g_). When *d*_w_ ≈ *d*_g_, pore fluctuations substantially modulate the potential energy experienced by the molecule, leading to mass-dependent diffusivity and thus mass selectivity. In contrast, when *d*_w_ ≫ *d*_g_, pore fluctuations induce only weak modulation of the potential energy, resulting in reduced mass selectivity. When *d*_w_ ≪ *d*_g_, the transport of both species is strongly suppressed, yielding little or no selectivity. **b** Diffusion over a static energy barrier. In the absence of barrier fluctuations, isotopologue selectivity follows the static mass-scaling limit. **c** Diffusion over fluctuating energy barriers. The stochastic modulation of the barrier height enables resonant activation, such that barrier-crossing events preferentially occur during transient low-barrier intervals. Because lighter particles traverse the low-barrier state more rapidly, these fluctuations amplify subtle mass differences into pronounced kinetic selectivity, yielding mass-selective transport beyond the static mass-scaling limit.

Molecular transport within nanopores is ubiquitous in biological and industrial processes, underpinning essential functions from cellular signaling to chemical separation. However, transport poses major challenges when channel dimensions approach the molecular scale (Fig. 1a)^1–6^. In such environments, transport is governed not only by spatial modulations of pore geometry, such as constrictions^7–10^, but also by temporal fluctuations in pore structures^11,12^. Indeed, molecular dynamics simulations incorporating structural flexibility have demonstrated enhanced diffusivity of gas molecules in metal–organic frameworks (MOFs) and water molecules in carbon nanotubes^13–17^. These spatiotemporal structural modulations are pronounced in a wide range of systems, from solute diffusion in hydrogels to the selective transport of water through aquaporins^18,19^. Despite these insights, structural disorder and temporal fluctuations are entangled in complex systems. This coupling precludes isolating the specific role of temporal variations, i.e., nanopore dynamics, in molecular transport, hindering the rational design of transport properties based on dynamic control beyond static structural optimization.

To address these complexities, MOFs with well-defined architectures provide opportunities to disentangle structural disorder from temporal fluctuations^20–27^. Among them, soft porous crystals (SPCs) are materials that can precisely control molecular diffusion through tunable nanoscale structural flexibility^20,28,29^. Recent experiments have shown that such materials exhibit marked kinetic selectivity even between molecules with nearly indistinguishable physicochemical properties, including water isotopologues^30,31^. Given that quantum tunneling and quantum sieving^32–34^ are unlikely to play a dominant role in these molecular separations at room temperature^35–37^ and that conventional transport theories based on static properties fail to account for these observations, these findings strongly suggest that intrinsic nanopore dynamics within SPCs play a pivotal role in selective separation. However, establishing a comprehensive theoretical framework to capture the underlying microscopic mechanisms remains a major challenge^37^.

Here, by establishing a minimal yet general and predictive theoretical framework, we elucidate how energy fluctuations generated by nanopore dynamics govern the selective transport of molecules with near-identical physicochemical properties and subtle mass differences. We demonstrate that diffusion on a fluctuating periodic energy landscape is maximized at a specific fluctuation rate. While resonant activation (RA)—the acceleration of barrier-crossing dynamics on a single fluctuating potential—has been extensively studied^38–44^, diffusion on a fluctuating periodic landscape governing nanopore transport remains largely unexplored (Fig. 1b). Crucially, this optimal rate exhibits mass dependence, enabling specific nanopore dynamics to preferentially accelerate one species over another and achieve transport selectivity that surpasses the static mass-scaling limit. As a benchmark for our framework, we investigate the selective diffusion of water isotopologues in two representative SPCs: [Cu(IDB)] (IDB = 5-(iminodibenzyl)isophthalate) and [Cu(OPTz)] (OPTz = 5-(5,5-dioxido-10H-phenothiazin-10-yl)isophthalate)^30,31,36^. Although the near-identical molecular dimensions and interaction potentials of water isotopologues make their separation challenging via conventional static design rules, [Cu(IDB)] exhibits pronounced selectivity, with H_2_O diffusing more than twice as fast as D_2_O, whereas [Cu(OPTz)] shows no such enhancement. By integrating our framework with quantum-chemical energy landscapes, we identify two key factors governing selectivity: the magnitude of energy-barrier fluctuations and the alignment between nanopore dynamics and the energy-fluctuation rate that maximizes selectivity. Collectively, these results establish a robust theoretical foundation for mass-selective transport in fluctuating nanoporous materials and offer design principles for tuning separation performance through intrinsic dynamics or external stimuli.

## Results

### Energy landscapes and gating energetics in [Cu(IDB)] and [Cu(OPTz)]

**Fig. 2:**
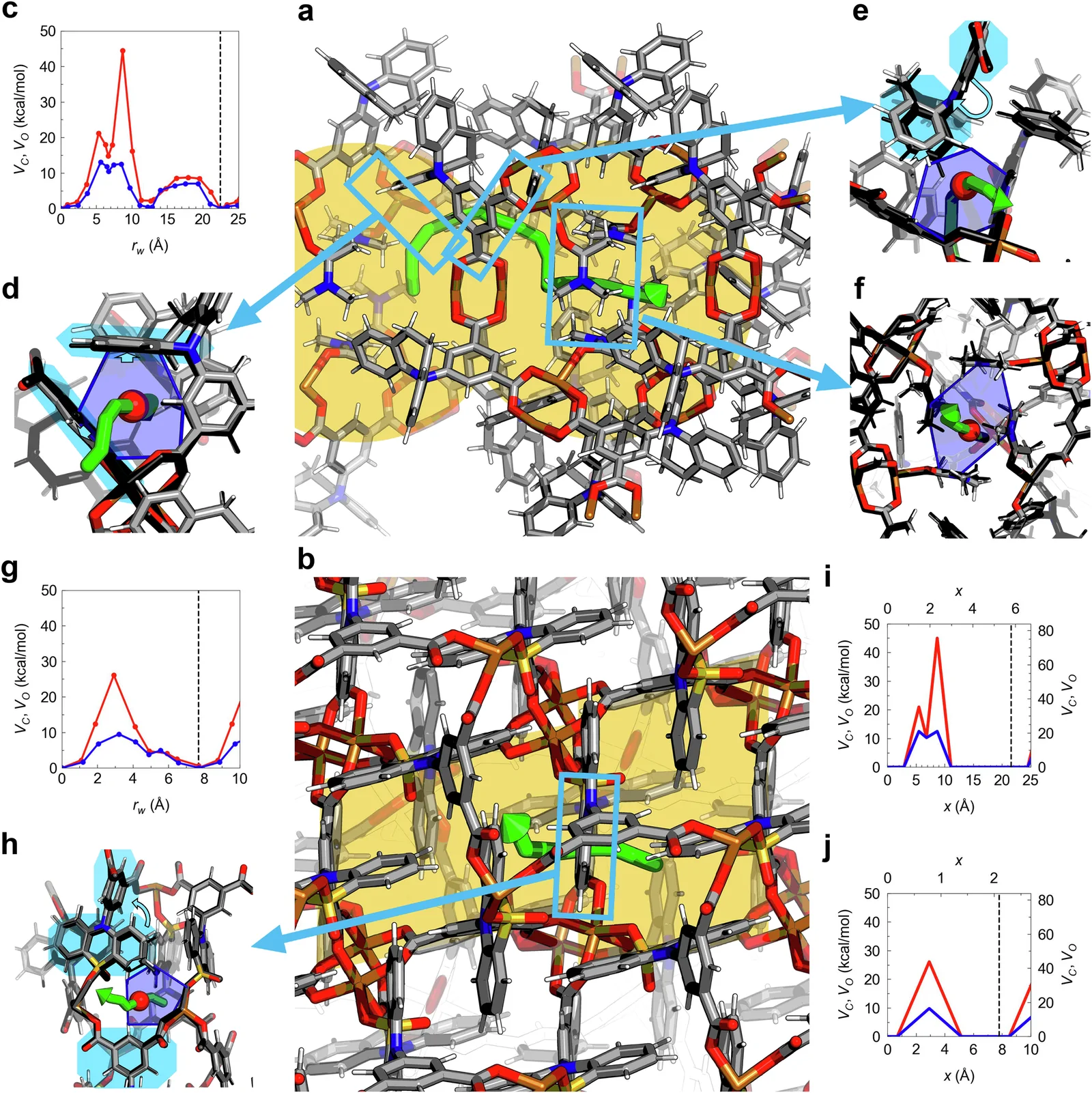
Water diffusion pathways, gate structures, and potential-energy profiles of [Cu(IDB)] and [Cu(OPTz)]. Diffusion pathways of a water molecule in the optimized structures of [Cu(IDB)] (**a**) and [Cu(OPTz)] (**b**). **c** Potential energy profiles along the closed (red) and open (blue) pathways in [Cu(IDB)]. Gate structures corresponding to the first (**d**), second (**e**), and third (**f**) energy barriers in [Cu(IDB)]. **g** Potential energy profiles along the closed (red) and open (blue) pathways in [Cu(OPTz)]. **h** Gate structure corresponding to the energy barrier of [Cu(OPTz)]. **i** and **j**, Simplified model potential energy profiles constructed for the theoretical modeling of [Cu(IDB)] (**i**) and [Cu(OPTz)] (**j**). In **a** and **b**, green arrows represent water diffusion pathways, and orange regions represent two cavities in the SPCs. Carbon, hydrogen, oxygen, nitrogen, sulfur, and copper atoms are shown in gray, white, red, blue, yellow, and orange, respectively. In **c**, **g**, **i**, and **j**, dashed vertical lines indicate boundaries of the periodic potential. In **d**–**f** and **h**, green arrows represent water diffusion pathways through gates. In **d**, **e**, and **h**, open-pathway structures are highlighted in color, whereas closed-pathway structures are rendered in black; ligand moieties contributing to gating are shown in cyan, and cyan arrows denote their dominant displacements from the closed to the open state. In **d**–**f**, and **h**, semi-transparent blue planes indicate the bottleneck regions along the water diffusion pathways. Parameters used to construct the model potentials are summarized in Supplementary Table 1.

To elucidate the microscopic origin of gating, we characterized the potential energy landscapes of water diffusion in the two SPCs. Starting from the reported structures^30,31^, we optimized the water-SPC framework systems using quantum-chemical calculations by placing a water molecule at the vacant Cu binding site in [Cu(IDB)] and at the cage center in [Cu(OPTz)]. Using these optimized geometries as initial states, we determined the water diffusion pathway under a fixed framework geometry (defined as the closed pathway) via the climbing-image nudged elastic band (CI-NEB) method (Fig. 2a, b)^45^. This method identifies the minimum-energy path (MEP) by optimizing a series of intermediate configurations while driving the highest-energy image to the saddle point. Subsequently, the corresponding open pathway was defined by fully relaxing the framework atoms for each image along the closed pathway (see “Methods”).

The calculated potential energy profiles along the open and closed pathways in [Cu(IDB)] reveal three distinct energy barriers at 5.5, 8.7, and 15–20 Å (Fig. 2c). The first barrier at 5.5 Å originates from steric hindrance between two IDB ligands (highlighted in cyan in Fig. 2d) and the water molecule. Upon transition from the open to the closed state, the center-to-center distance between these ligands shortens by ~0.4 Å, raising the barrier height from 12.5 to 21.2 kcal mol^−1^. The second barrier at 8.7 Å arises from the constriction of the pore aperture by the iminodibenzyl group. During the transition from the open to the closed form, the dihedral angle between the iminodibenzyl and isophthalate groups (highlighted in cyan in Fig. 2e) decreases from 21° to 11.5°, markedly increasing the barrier height from 12.5 to 45.0 kcal mol^−1^ (Supplementary Movie 1). The third barrier, attributed to the spatial congestion caused by four dimethylacetamide molecules (Fig. 2f), was excluded from subsequent analyses because it is independent of ligand fluctuations.

In contrast, [Cu(OPTz)] exhibits a single energy barrier arising from steric hindrance at the gate site formed by the 5,5-dioxido-10H-phenothiazin-10-yl and isophthalate groups (Fig. 2g). An increase in the bending angle between these groups (highlighted in cyan in Fig. 2h) from 54.5° to 57.3° narrows the water pathway and consequently raises the barrier height from 9.5 to 26.1 kcal mol^−1^ (Supplementary Movie 2).

These results indicate that both SPCs possess gated architectures that maintain localized energy barriers, even in their open states. Crucially, nanopore dynamics—structural fluctuations of the flexible gate-forming ligands—substantially elevate the energy barriers in the closed state by ~10 and ~33 kcal mol^−1^ for the first and second barriers of [Cu(IDB)], respectively, and ~16 kcal mol^−1^ for [Cu(OPTz)]. As demonstrated below, this profound energy gap between the open and closed pathways is a key factor in the fluctuation-driven selectivity of dynamic nanopores.

### Theoretical framework for mass-selective transport in dynamic nanopores

The quantum-chemical calculations indicate that the dominant structural changes controlling transport are localized at each barrier in these SPCs, suggesting that these gating events are effectively independent. Based on this locality, we model three-dimensional water diffusion within the SPC as an effective one-dimensional (1D) process on a stochastically fluctuating periodic potential. Because pore dimensions are comparable to the size of a water molecule, water transport falls within the Knudsen regime, in which frequent collisions with walls dominate molecular motion^1–5^. Under these conditions, because the timescale for crossing high-energy barriers is significantly longer than that for momentum relaxation, we employ a coarse-grained description based on the overdamped Langevin equation. This framework incorporates Knudsen-type mass scaling of the self-diffusion coefficient through the fluctuation-dissipation theorem (see Methods). We represent nanopore dynamics as symmetric stochastic switching between the open and closed potential-energy landscapes, with identical fluctuation rates in both directions. The landscapes are described by piecewise-linear functions derived from quantum-chemical calculations (Fig. 2i, j).

All diffusion coefficients calculated in the present framework are self-diffusion coefficients, *D*_*s*_. This is because the model describes the stochastic motion of a single particle on a fluctuating energy landscape and does not explicitly include guest–guest interactions, excluded-volume effects, or loading-dependent changes in the effective potential. Thus, the framework corresponds to the dilute-loading limit, where guest–guest interactions are negligible, and *D*_*s*_ coincides with the transport diffusion coefficient *D*_*t*_^2,4,5^. Accordingly, the present framework describes self-diffusion and provides the dilute-limit diffusivity component of transport selectivity. At finite loading, although the cavities of [Cu(IDB)] and [Cu(OPTz)] accommodate only a limited number of water molecules, estimated to be at most approximately five and six, respectively^31^, water–water interactions and associated collective and thermodynamic effects may modify *D*_*t*_. Quantitative treatment of such effects will require future atomistic simulations that explicitly include guest–guest interactions and realistic pore geometry.

### Fluctuation-amplified mass selectivity

**Fig. 3:**
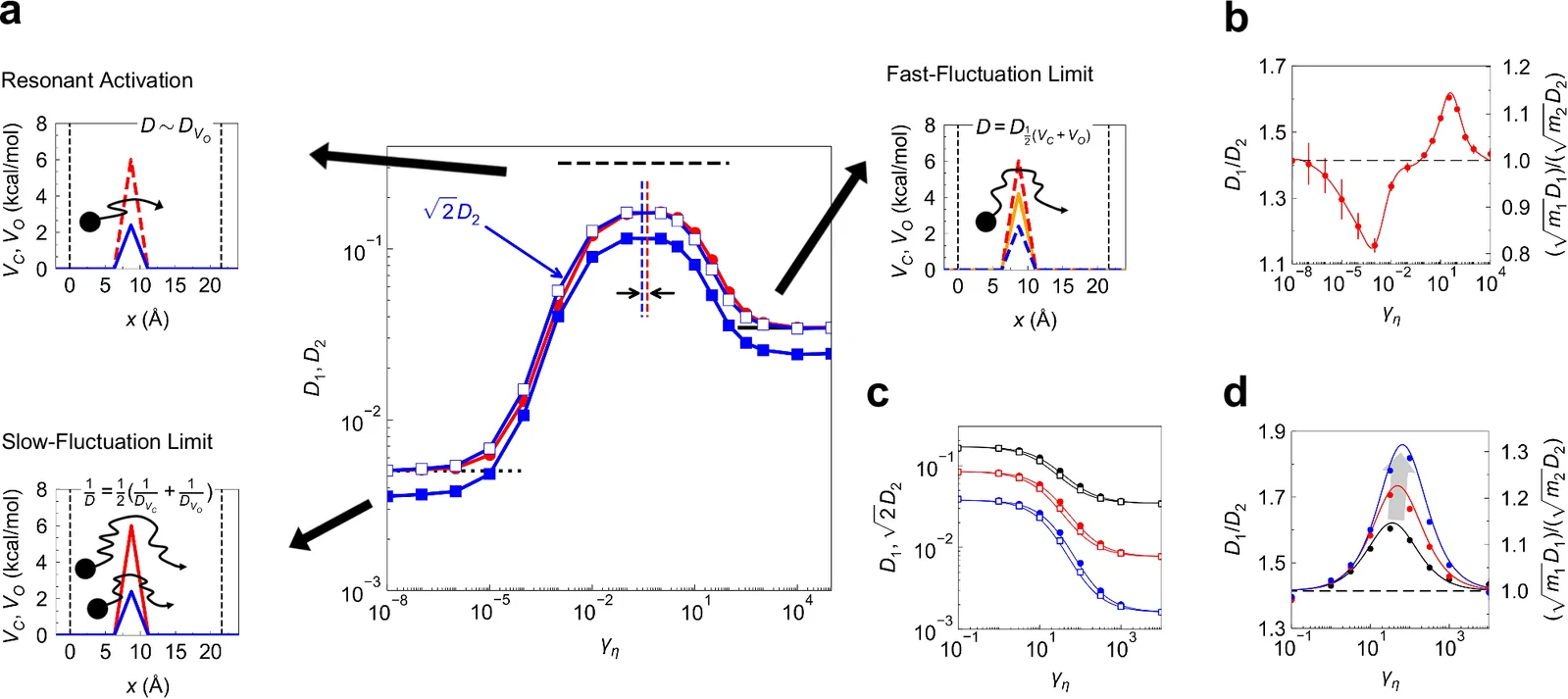
Resonant activation and its influence on self-diffusion coefficients and selectivity. **a** Self-diffusion coefficients *D*_1_ (filled red circles) and *D*_2_ (filled blue squares) for particles with masses *m*_1_ = 1 and *m*_2_ = 2, respectively, along with $\sqrt{2}$*D*_2_ (open blue squares). Results were obtained using the model shown in Supplementary Fig. 1a with barrier heights (*V*_*C*_, *V*_*O*_) = (6.0, 2.4) kcal mol^−1^. The profile of *D*_2_ is systematically shifted relative to *D*_1_ by a factor of $\sqrt{2}$, as evidenced by the alignment between *D*_1_ and $\sqrt{2}$*D*_2_ (open blue squares). Solid and dotted black lines indicate analytical *D*_1_ values in the fast- and slow-fluctuation limits, respectively, whereas the dashed black line denotes the *D*_1_ value for the open pathway. Three surrounding insets illustrate the effective potentials and barrier-crossing mechanisms: in the fast-fluctuation limit (top right), the particle (black circle) experiences an averaged potential (orange line); in the RA regime (top left), transport is dominated by the lower energy-barrier pathway (blue line); and in the slow-fluctuation limit (bottom left), particles stochastically cross either the high- or low-energy barrier. **b** Dependence of selectivity (*D*_1_/*D*_2_) on *γ*_*η*_. **c** Self-diffusion coefficients *D*_1_ (circles) and $\sqrt{2}$*D*_2_ (squares) for three systems with (*V*_*C*_, *V*_*O*_) = (6.0, 2.4) (black), (7.5, 3.0) (red), and (9.0, 3.6) (blue) kcal mol^−1^, shown in Supplementary Fig. 1a. **d** Dependence of selectivity on *γ*_*η*_ for the three systems shown in (**c**). In **b** and **d**, error bars represent standard deviations estimated using the bootstrap method, and dashed lines represent the static mass-scaling limit.

To clarify the effect of energy-barrier fluctuations on long-time transport, we first analyzed the dependence of the self-diffusion coefficient on the energy-fluctuation rate *γ*_*η*_. Figure 3a shows the self-diffusion coefficient *D*_1_ (red curve) for a particle with mass *m*_1_ = 1 as a function of *γ*_*η*_. In the fast-fluctuation limit (*γ*_*η*_ → ∞), the particle effectively experiences the average potential of the two pathways, and *D*_1_ approaches the self-diffusion coefficient corresponding to this average landscape (black solid line and fast-fluctuation limit panel in Fig. 3a). In the slow-fluctuation limit (*γ*_*η*_ → 0), the potential switching rate is much slower than the diffusive traversal of one period in the open and closed states. In this regime, the effective self-diffusion coefficient is determined by the average traversal time across one period. Because the open and closed states are equally populated in our model, *D*_1_ can be expressed as the harmonic mean of the self-diffusion coefficients of the two pathways (black dotted line and slow-fluctuation limit panel in Fig. 3a). Crucially, *D*_1_(*γ*_*η*_) does not vary monotonically between these limits; instead, it exhibits a pronounced maximum at approximately *γ*_*η*_ ≈ 0.4, reflecting accelerated transport assisted by energy fluctuations, where barrier-crossing events are preferentially facilitated during transient intervals in the low-barrier open state^38^ (resonant activation panel in Fig. 3a). The acceleration of barrier crossing driven by fluctuations has been extensively studied for a single barrier^38–44^; here we establish transport enhancement for long-time diffusion on a fluctuating periodic landscape, which is directly relevant to nanopore transport. This result provides a rational basis for understanding molecular transport through dynamic nanopores. Furthermore, this enhanced diffusion is reminiscent of the cooperative gate-opening motion observed in two-dimensional dynamic porous matrices^12^.

Next, to examine the mass dependence, we evaluated the self-diffusion coefficient *D*_2_(*γ*_*η*_) for a model system with a heavier particle (*m*_2_ = 2). As shown in Fig. 3a, *D*_2_(*γ*_*η*_) exhibits an RA behavior similar to that of *D*_1_(*γ*_*η*_). Assuming Knudsen diffusion, in which the self-diffusion coefficient scales with *m*^−1/2^, *D*_1_ and $\sqrt{{m}_{2}/{m}_{1}}$*D*_2_ coincide in the fast- and slow-fluctuation limits, as well as the plateau region within the RA regime. However, a clear discrepancy emerges: the entire profile of *D*_2_(*γ*_*η*_) is systematically shifted toward lower *γ*_*η*_ by a factor of $\sqrt{{m}_{2}/{m}_{1}}$ relative to *D*_1_(*γ*_*η*_), reflecting the different intrinsic timescales of the two particles. Consequently, the *D*_1_/D_2_ ratio, hereafter referred to as the selectivity, exceeds the static mass-scaling limit of $\sqrt{{m}_{2}/{m}_{1}}$ when *γ*_*η*_ > 0.7, reaching a maximum at *γ*_*η*_ ≈ 40 (Fig. 3b). This enhancement demonstrates that energy fluctuations preferentially accelerate the barrier-crossing dynamics of the lighter particle in the intermediate regime where the fluctuation rate matches its characteristic crossing time. Conversely, at lower *γ*_*η*_ (e.g., *γ*_*η*_ ≈ 10^−4^), the motion of the lighter particle is relatively suppressed, reducing the selectivity below the static limit. These results reveal that energy-barrier fluctuations can amplify subtle mass differences into significant selectivity.

To gain mechanistic insights into mass selectivity in diffusion, we investigated the dependence of self-diffusion coefficients on the energy-barrier height. Using three model systems with varying barrier heights (Supplementary Fig. 1a), we calculated the self-diffusion coefficients *D*_1_(*γ*_*η*_) and $\sqrt{2}$*D*_2_(*γ*_*η*_) for particles with masses *m*_1_ = 1 and *m*_2_ = 2 (Fig. 3c). Our analysis focuses on the regime *γ*_*η*_ > 10^−1^, where the selectivity exceeds the static limit of $\sqrt{2}$. As discussed previously, the self-diffusion coefficient in the fast-fluctuation limit is determined by the average potential energy of the open and closed pathways. In contrast, at the RA peak, transport is governed by the low-barrier open pathway. Therefore, elevating the energy barriers of both the closed *V*_*C*_(*x*) and open *V*_*O*_(*x*) pathways amplifies the contrast between the self-diffusion coefficients in the fast-fluctuation and RA regimes, thereby enhancing isotopologue selectivity (Fig. 3d). Furthermore, increasing the energy gap between *V*_*C*_(*x*) and *V*_*O*_(*x*) shifts the selectivity peak toward higher *γ*_*η*_ values. As the *V*_*C*_/*V*_*O*_ ratio increases, barrier crossing in the closed state is exponentially suppressed, and successful crossing becomes progressively restricted to transient intervals in the open state. Although increasing *γ*_*η*_ shortens the duration of each open-state interval, it increases the frequency with which the particle encounters the open state^43,44^. Because the lighter particle has the shorter characteristic crossing time, it can effectively exploit these shorter open-state intervals even at higher *γ*_*η*_, whereas the heavier particle fails to complete its crossing before the barrier rises again. This mass-dependent matching between the gate lifetime and particle crossing time shifts the selectivity peak toward higher *γ*_*η*_ values as the energy gap increases.

**Fig. 4:**
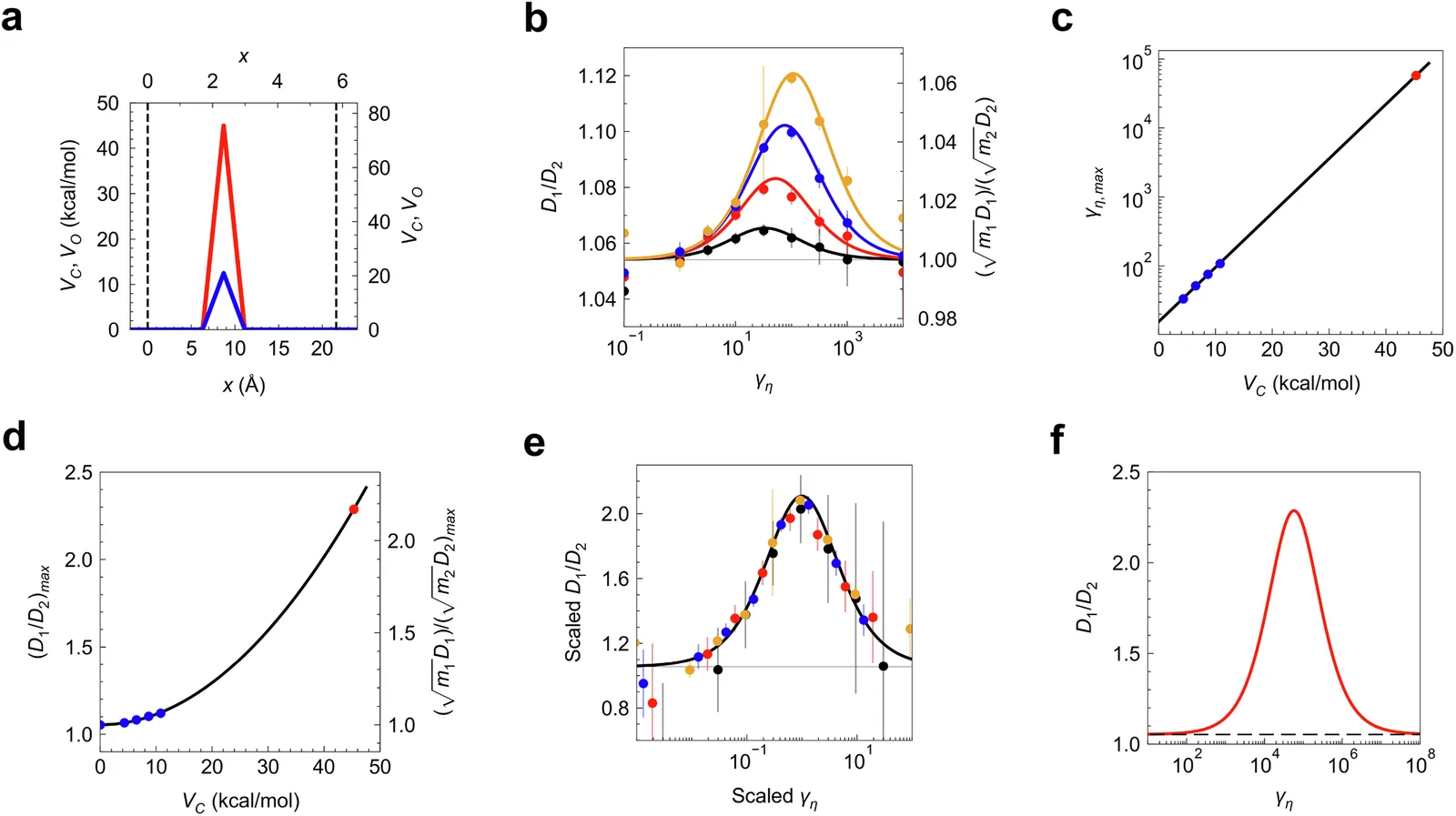
Scaling behavior and extrapolation of selectivity in the single-barrier [Cu(IDB)] model. **a** A single-barrier model representing the second peak in [Cu(IDB)], with barrier heights (*V*_*C*_, *V*_*O*_) of (45.0, 12.5) kcal mol^−1^, corresponding to the second energy barrier identified for [Cu(IDB)] (Fig. 2i). The parameters of the scaled-down systems for this model are listed in the first row of Supplementary Table 2. **b** Dependence of selectivity on *γ*_*η*_ for particles with masses *m*_1_ = 1 and *m*_2_ = 20/18 in four low-barrier systems (see Supplementary Table 2). Peak positions *γ*_*η,max*_ (**c**) and peak heights (*D*_1_/*D*_2_)_*max*_ (**d**) as a function of the closed-pathway barrier height *V*_*C*_. Blue dots indicate calculated values for the low-barrier systems, whereas red dots represent extrapolated values for the original high-barrier system. Black curves represent fits to the calculated data; coefficients of determination (*R*^2^) are 0.998 (**c**) and 0.990 (**d**). **e** Dependence of the scaled selectivity on *γ*_*η*_, demonstrating the collapse of data from all four systems onto a single universal curve (black line). Standard deviations are scaled accordingly. **f** Dependence of selectivity on *γ*_*η*_ for particles with masses *m*_1_ = 1 and *m*_2_ = 20/18 in the model presented in (**a**). In **b** and **e**, error bars represent standard deviations estimated using the bootstrap method.

We further examined the *γ*_*η*_ dependence of the isotopologue selectivity for H_2_O (*m*_1_ = 1) and D_2_O (*m*_2_ = 20/18) in the single-barrier [Cu(IDB)] model, using high barriers derived from quantum-chemical calculations (*V*_*C*_ = 45.0 and *V*_*O*_ = 12.5 kcal mol^−1^; Fig. 4a). Because a direct numerical evaluation of *D*_1_/*D*_2_ under such high-barrier conditions is computationally prohibitive, we estimated the selectivity by extrapolating results from four systems with systematically reduced barriers (see Supplementary Note 1 and Supplementary Table 2). As shown in Figs. 4b–d, the peak value and position of the selectivity exhibit a systematic dependence on the barrier height *V*_*C*_. Importantly, the collapse of these data onto a single master curve reveals a universal scaling law governing this process: fluctuations drive isotopologue selectivity beyond the static mass-scaling limit, regardless of the barrier height (Fig. 4e). This scaling behavior indicates that the underlying mechanism is invariant with respect to barrier height, providing evidence of generality. The extrapolated maximum selectivity reaches 2.29, more than twice the static mass-scaling limit ($\sqrt{{m}_{2}/{m}_{1}}$ ≈ 1.05) (Fig. 4f). This enhanced selectivity originates from the amplified energy gap between the two pathways. These results demonstrate that even for water isotopologues with subtle mass differences, large energy fluctuations can amplify selectivity far beyond the static mass-scaling limit.

### Fluctuation-engineered selectivity in multi-barrier landscapes

**Fig. 5:**
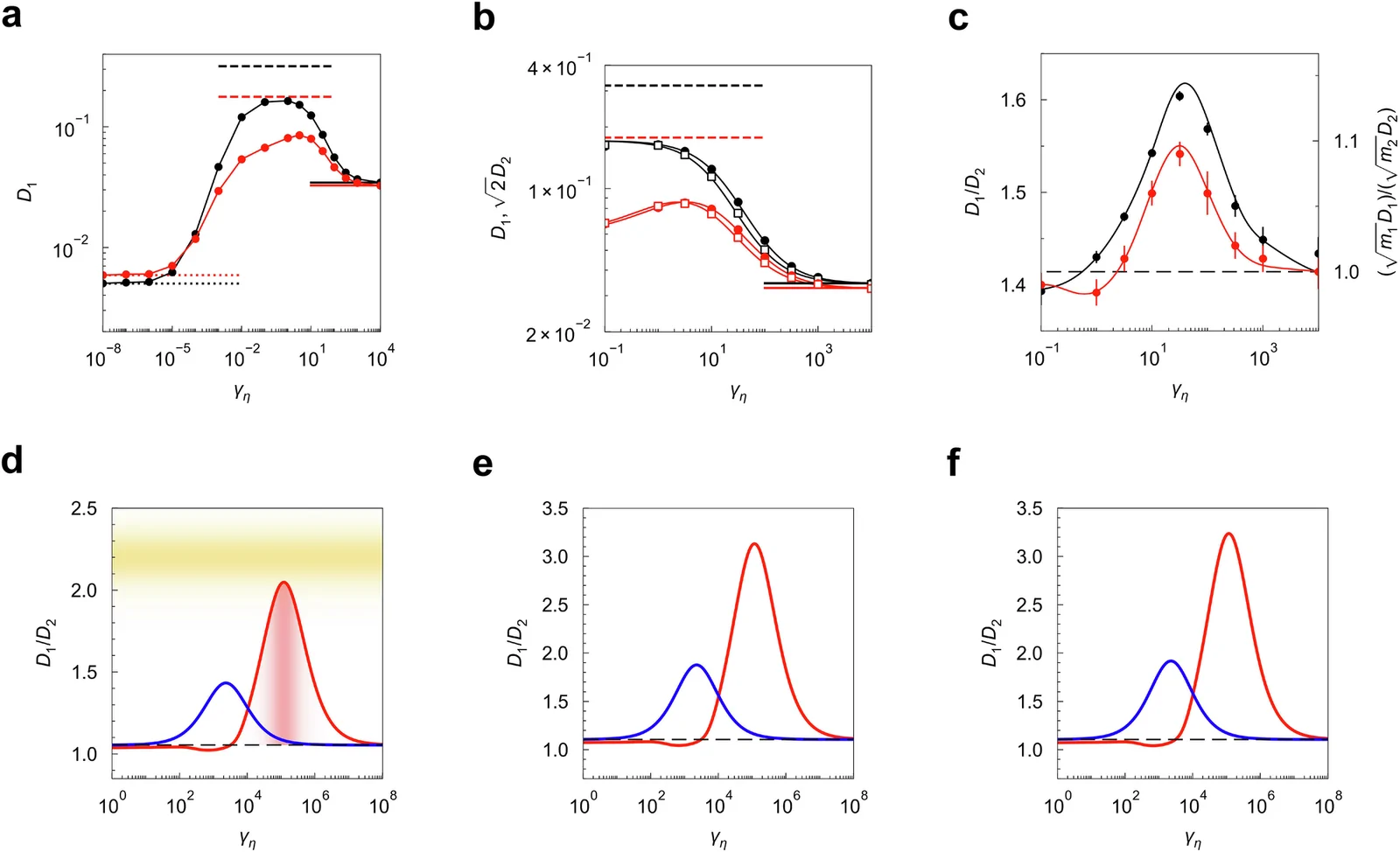
Dependence of the self-diffusion coefficient and isotopologue selectivity on *γ*_*η*_. **a** Self-diffusion coefficient *D*_1_ for a particle with mass *m* = 1 in single- (black) and double-barrier (red) models. The single-barrier model uses barrier heights (*V*_*C*_, *V*_*O*_) = (6.0, 2.4) kcal mol^−1^. The double-barrier model incorporates barrier peaks at *x* = 5.5 Å (*V*_*C*_ = 3.6, *V*_*O*_ = 2.4 kcal mol^−1^) and *x* = 8.7 Å (*V*_*C*_ = 6.0, *V*_*O*_ = 2.4 kcal mol^−1^). **b** Self-diffusion coefficients *D*_1_ (*m*_1_ = 1; filled circles) and $\sqrt{2}$*D*_2_ (*m*_2_ = 2; open squares) for both models. **c** Selectivity of the lighter isotopologue (*m*_1_ = 1) over the heavier isotopologue (*m*_2_ = 2) for the two models. **d** Selectivity of H_2_O over D_2_O for [Cu(IDB)] (red) and [Cu(OPTz)] (blue). The red shaded region highlights the range of *γ*_*η*_ over which *D*_1_/*D*_2_ > 1.6 for [Cu(IDB)], whereas it remains below 1.1 for [Cu(OPTz)]. The orange shaded region represents the selectivity estimated from measurements by Su et al.^31^. **e** Selectivity of H_2_O over T_2_O for [Cu(IDB)] (red) and [Cu(OPTz)] (blue). **f** Selectivity of H_2_O over D_2_O, assuming a stronger mass dependence (*D* ∝ *m*^−1^). In **a** and **b**, solid, dotted, and dashed lines represent theoretical *D*_1_ values in the fast- and slow-fluctuation limits and for the open pathway, respectively. In **c**, error bars represent standard deviations estimated using the bootstrap method. In **c**–**f**, dashed lines represent the static mass-scaling limit.

Next, we examined diffusion in a more complex double-barrier landscape mimicking the potential energy profile of [Cu(IDB)], in which two energy barriers exist along both the open and closed pathways (Supplementary Fig. 1b). Figure 5a compares *D*_1_(*γ*_*η*_) for a particle of mass *m*_1_ = 1 in this system with that in the simpler single-barrier model (Supplementary Fig. 1a). Although the *D*_1_ values in both systems are comparable at the fast- and slow-fluctuation limits, the double-barrier system exhibits a diminished enhancement of *D*_1_ via RA compared to the single-barrier case. This attenuation occurs because the spatially extended low-barrier region in the double-barrier landscape reduces the relative contribution of the RA effect to global transport.

To evaluate the effect of topological complexity in the energy landscape on selective separation, we compared the profiles of *D*_1_(*γ*_*η*_), $\sqrt{2}$*D*_2_(*γ*_*η*_), and the selectivity *D*_1_/*D*_2_ between the single- and double-barrier models (Fig. 5b and c). As predicted in Fig. 5a, the maximum selectivity is reduced in the double-barrier model (Fig. 5c) because the RA-driven enhancement is less pronounced than that in the single-barrier model. However, consistent with the single-barrier results (Fig. 3d and Supplementary Fig. 2a), widening the energy gap between *V*_*C*_(*x*) and *V*_*O*_(*x*) within the double-barrier system amplifies selectivity and shifts the peak toward higher *γ*_*η*_ values (Supplementary Fig. 2b). This finding confirms that the fundamental mechanism of RA persists in complex energy landscapes. Collectively, these results demonstrate that tuning the energy-fluctuation rate can selectively facilitate the diffusion of the lighter isotopologue over the heavier one, regardless of landscape complexity.

### Mechanistic origin of contrasting isotopologue selectivity between [Cu(IDB)] and [Cu(OPTz)]

We next investigated water isotopologue selectivity using the double-barrier model for [Cu(IDB)] (Fig. 2i) and the single-barrier model for [Cu(OPTz)] (Fig. 2j), with energy-barrier heights determined from quantum-chemical calculations. Because the high-energy barriers in these systems preclude the direct evaluation of self-diffusion coefficients, we employed the extrapolation scheme described above (Supplementary Fig. 3). As shown in Fig. 5d, the [Cu(IDB)] model reaches a pronounced selectivity of 2.05 at *γ*_*η*_ = 1.18 × 10^5^, nearly twice the static mass-scaling limit. This result, obtained from quantum-chemical energy barriers with no adjustable parameters, is quantitatively consistent with the experimental selectivity ( > 2)^31^, demonstrating the predictive capability of our framework. In contrast, the [Cu(OPTz)] model yields a maximum selectivity of only 1.43 (at *γ*_*η*_ = 2.3 × 10^3^). This disparity in selectivity indicates that large-amplitude energy-barrier fluctuations in [Cu(IDB)] substantially amplify isotopologue-selective diffusion, whereas smaller-amplitude fluctuations in [Cu(OPTz)] yield only limited enhancement. Extending this analysis to the heavier isotopologue T_2_O (*m*_2_ = 22/18) yields higher selectivity (Fig. 5e), with the selectivity of H_2_O over T_2_O reaching 3.14 for [Cu(IDB)] compared to 1.88 for [Cu(OPTz)]. This fluctuation-induced selectivity remains robust, irrespective of the underlying mass-scaling laws. For H_2_O and D_2_O, the selectivity in the [Cu(IDB)] model reaches 3.26 under *m*^−1^ scaling, significantly exceeding the 2.05 obtained with *m*^−1/2^ scaling (Fig. 5f). These results demonstrate that fluctuation-driven selectivity is a general phenomenon that persists regardless of specific mass-scaling assumptions or energy landscape geometries, establishing it as a fundamental mechanism for efficient isotope separation.

Our analysis reveals that the optimal energy-fluctuation rate for maximizing selectivity differs by ~50-fold between the two SPCs (Fig. 5d). This optimal rate is determined by the magnitude of energy fluctuations, namely, the energy gap, and does not necessarily coincide with the intrinsic timescales of nanopore dynamics that induce these fluctuations. Consequently, maximum selectivity emerges only when the characteristic timescale of the nanopore dynamics aligns with the optimal fluctuation rate determined by the energy landscape. To evaluate the timescales of gating-induced dynamics in the two SPCs, we performed normal mode analysis (NMA) on the structural transitions between the open and closed states. NMA revealed that the vibrational frequencies of the modes contributing to gating are comparable for both SPCs, falling within the range of ~15 − 40 cm^−1^ (Supplementary Movies 3–11). Given the marked contrast in selectivity between the two SPCs, the effective gating rate relevant to the experiments is estimated to lie within the range of 2 × 10^4^ to 8 × 10^5^. Within this regime, [Cu(IDB)] maintains a high selectivity (*D*_1_/*D*_2_ > 1.6), whereas [Cu(OPTz)] exhibits negligible selectivity (*D*_1_/*D*_2_ < 1.1). These results suggest that although both materials possess gating modes with similar intrinsic frequencies, the effective gating rate of [Cu(IDB)] is aligned with the optimal range for separation, whereas that of [Cu(OPTz)] deviates significantly from this range. Due to the high-energy barriers, precisely determining the effective friction coefficient *γ*_*eff*_ and energy-fluctuation rate *γ*_*η*_ through fully atomistic simulations remains computationally prohibitive. However, assuming that ligand motions are described by a Langevin equation with a friction coefficient *γ*_*eff*_, the energy-fluctuation rate *γ*_*η*_ can be approximated by *γ*_*eff*_ based on established vibrational relaxation theories^46,47^. Under this approximation, the optimal rate *γ*_*η*_ likely falls within the sub-picosecond timescale, consistent with the normal modes intrinsic to these SPCs.

We also estimated the self-diffusion coefficient of H_2_O, *D*_1_, at the fluctuation rate that maximizes selectivity for the high-barrier systems shown in Figs. 4f and 5d–f. Because direct numerical evaluation of *D*_1_ is computationally prohibitive for these high-barrier systems, *D*_1_ was evaluated using an extrapolation approach (Supplementary Note 2, Supplementary Fig. 4, and Supplementary Table 3). For the single-barrier [Cu(IDB)] model shown in Fig. 4f, *D*_1_ is estimated to be ~2.3×10^−20^ *m*^2^ *s*^−1^. For the systems shown in Fig. 5d, *D*_1_ is ~1.2 × 10^−20^ *m*^2^ *s*^−1^ for [Cu(IDB)] and ~1.2×10^−15^ *m*^2^ *s*^−1^ for [Cu(OPTz)]. The corresponding values for Fig. 5e, f are summarized in Supplementary Table 3. These small self-diffusion coefficients, particularly for [Cu(IDB)], reflect the high-energy barriers associated with water diffusion through gated SPCs. Importantly, however, in this regime, dynamic barrier modulation can strongly amplify the subtle mass difference between H_2_O and D_2_O, allowing the self-diffusion ratio to exceed the static mass-scaling limit. Thus, the small self-diffusivities and large selectivities are not contradictory; rather, they are two consequences of the same high-barrier, fluctuation-assisted transport mechanism. Consistent with the recent discussion on diffusion anomalies in nanopores^48^, these self-diffusion coefficients provide quantitative reference points for future experimental and computational tests of fluctuation-assisted transport in dynamic nanopores.

Together, these results demonstrate the predictive capability of the present framework. Using quantum-chemical energy landscapes without adjustable parameters, the model quantitatively reproduces the large H_2_O/D_2_O selectivity observed in [Cu(IDB)] while explaining the weaker enhancement in [Cu(OPTz)]. Importantly, the analysis identifies two transferable design principles for dynamic nanopores: the magnitude of energy-barrier fluctuations between open and closed pathways and the alignment between the intrinsic nanopore dynamics and the energy-fluctuation rate that maximizes selectivity. These findings suggest that molecular transport properties can be rationally designed and controlled by actively manipulating system dynamics, either by tailoring intrinsic dynamics through ligand substitution or by applying external stimuli such as electric fields.

## Discussion

In this study, we elucidated the fundamental mechanism underlying mass selectivity within dynamically fluctuating nanoporous environments by establishing a minimal yet general and predictive theoretical framework. We demonstrated that energy-barrier fluctuations generated by nanopore dynamics can accelerate diffusion and amplify subtle mass differences into pronounced kinetic disparities within molecular-sized nanopores, yielding separations that surpass the static mass-scaling limit. By integrating our framework with the quantum-chemical energy landscapes of [Cu(IDB)] and [Cu(OPTz)], we provided a unified explanation for the contrasting isotopologue selectivities observed in these SPCs without introducing any adjustable parameters. This analysis further established that the fluctuation-amplification mechanism persists across both single- and double-barrier landscapes and is therefore not restricted to a specific system or energy-landscape geometry. Furthermore, we identified two factors governing selectivity: the magnitude of energy-barrier fluctuations between open and closed pathways and the alignment between the characteristic timescale of nanopore dynamics and the optimal energy-fluctuation rate for selectivity. These results establish nanopore dynamics as a controllable design dimension for transport, complementary to conventional static design, and suggest concrete strategies for actively tuning transport by engineering nanopore dynamics through ligand substitution or external stimuli such as electric fields. More broadly, the mechanism uncovered here extends beyond isotopologues to the mass-selective transport of species with nearly identical interaction properties but different masses in fluctuating nanoporous environments. Overall, this study reveals that structural fluctuations are not merely a source of complexity but can also serve as a functional tool for actively tailoring mass-selective transport in dynamic nanopores.

## Methods

### Quantum-chemical calculations of gating energetics and diffusion pathways

All quantum-chemical calculations were performed using Quantum Espresso 7.3^49^. For both SPCs, structural optimization, CI-NEB calculations, and NMA were performed using the Perdew-Burke-Ernzerhof (PBE) functional with the projector augmented-wave (PAW) method, as previously reported^30,31^. The CI-NEB method was used to determine the MEP and the transition state between the initial and final configurations. In this method, intermediate images are connected by virtual spring forces to maintain path continuity, whereas the nudged force projection prevents relaxation toward local minima. The climbing image scheme further refines the highest-energy image to locate the saddle point^45^. Plane-wave basis sets with an energy cutoff of 639.2 eV were employed, together with Grimme’s semi-empirical D3 dispersion correction. Owing to the large unit cell sizes, Brillouin zone sampling was restricted to the Γ-point. Atomic coordinates were relaxed until the energy and force convergence thresholds were below 1.36 × 10^−8^ eV and 5.14 × 10^−5^ eV Å^−1^, respectively. Gaussian spreading with an energy value of 0.0272 eV was employed. A Hubbard *U* correction with a *U* value of 4.0 eV was applied to the localized *d* electrons of the Cu^2+^ center.

For [Cu(IDB)], the open and closed pathways exhibit three distinct energy barriers, requiring three separate CI-NEB calculations. Seven images were used for each segment, resulting in a total of 19 images along the path. For [Cu(OPTz)], the single-barrier pathway was evaluated using nine images. The force convergence threshold was 0.05 eV Å^−1^ for CI-NEB calculations.

NMA was performed at the second barrier of the open pathway for [Cu(IDB)] (Fig. 2e) and at the barrier along the open pathway for [Cu(OPTz)] (Fig. 2h). Hessian matrices were obtained using fourth-order finite differences of atomic forces with displacements of ±0.001 and ±0.002 Å for each coordinate.

### Theoretical modeling and simulations of fluctuation-driven diffusion

Water diffusion within SPCs is modeled as an effective 1D process on a time-fluctuating periodic potential. Because the SPC pores confine water molecules on the molecular scale, water molecules frequently collide with the pore walls (i.e., the Knudsen regime), leading to momentum relaxation. This relaxation is phenomenologically incorporated into the effective friction coefficient, *γ*_*eff*_. Because the energy barriers at the gating sites are sufficiently high, the timescale for barrier crossing is significantly longer than that for velocity relaxation, allowing the inertial term to be neglected. In the Knudsen regime, the self-diffusion coefficient *D*_*s*_ is proportional to *m*^−1/2^, where *m* is the particle mass. According to the fluctuation-dissipation theorem, because *D*_*s*_ is inversely proportional to *γ*_*eff*_, *γ*_*eff*_ scales as *m*^1/2^. Consequently, *γ*_*eff*_ is decomposed into a mass-dependent factor *m*^1/2^ and a mass-independent component *γ*_*eff*_ʹ. Assuming that the ligands at the gate sites fluctuate between the open and closed pathways, the diffusive behavior of a particle with mass *m* is described by the following overdamped Langevin equation:

1 $$\it \sqrt{{{\it{m}}}}\frac{{{\it{dx}}}}{{{\it{dt}}}}=-\left(\frac{{{\it{dV}}}{\rm(}{{\it{x}}}{\rm)}}{{{\it{dx}}}} + \frac{{{\it{d}}}\Delta {{\it{V}}}{\rm(}{\it{x}}{{\rm{)}}}}{{{\it{dx}}}}\eta {{\rm{(}}}t{{\rm{)}}}\right) + \xi{{\rm{(}}}t{{\rm{)}}}$$

Here, *x* denotes the particle position, and *t* represents the time variable scaled by *γ*_*eff*_ʹ (*t* = *t*ʹ/*γ*_*eff*_ʹ), where *t*ʹ is the real time. *V*(*x*) and Δ*V*(*x*) represent the average potential and potential difference between the open and closed pathways, respectively. *ξ*(*t*) is Gaussian white noise that satisfies the fluctuation-dissipation theorem, characterized by 〈*ξ*(*t*)〉 = 0 and 〈*ξ*(*t*)*ξ*(*s*)〉 = 2*k*_B_*Tδ*(*t* − *s*), where *k*_B_ is the Boltzmann constant, *T* is the temperature, and *δ*(*t*) is the Dirac delta function. The potential energy fluctuation in each periodic segment is modeled using dichotomous noise *η*(*t*). We assume that the energy-fluctuation rate *γ*_*η*_ from the closed to open pathway is equal to that of the reverse process. The statistical properties of *η*(*t*) are 〈*η*(*t*)〉 = 0 and 〈*η*(*t*)*η*(*s*)〉 = exp( − 2*γ*_*η*_ | *t*–*s* | ).

To implement the model, Eq. (1) was solved numerically using the overdamped version of the BAOAB method^50^,

2 $${x}_{n+1}={x}_{n}-\Delta {t}\frac{V^{\prime}({x}_{n})+\Delta V^{\prime}({x}_{n}){\eta}_{n}}{\sqrt{m}}+\sqrt{\Delta t}\frac{\left({\xi}_{n}+{\xi}_{n+1}\right)}{2\sqrt{m}}$$

where *x*_*n*_ denotes the particle position at the *n*-th step, and *V*ʹ and Δ*V*ʹ represent the gradients of *V* and Δ*V*, respectively. Simulations were performed in a scaled unit system, where the fundamental units for energy, mass, and time were defined as 300 K, 18 g mol^−1^, and 1 ps, respectively. In this unit system, the corresponding unit of length was 3.72 Å. The time step for numerical integration Δ*t* was set to 1×10^−4^. The implementation of this methodology is available on GitHub^51^.

To evaluate the MSD, we performed 256 independent simulation runs for each system. Each trajectory was evolved for at least 10^12^ steps starting from different initial conditions. The self-diffusion coefficient *D*_*s*_ was calculated from the MSD 〈Δ*x*^2^(*t*)〉 using the relation *D*_*s*_ = lim_*t*→∞_〈Δ*x*^2^(*t*)〉/2*t* (Supplementary Fig. 5).

## Supplementary information

Supplementary Information

Transparent Peer Review file

Description of Additional Supplementary Files

Supplementary movie 1

Supplementary movie 2

Supplementary movie 3

Supplementary movie 4

Supplementary movie 5

Supplementary movie 6

Supplementary movie 7

Supplementary movie 8

Supplementary movie 9

Supplementary movie 10

Supplementary movie 11

## Data Availability

All the data supporting the findings of this study are available within the article and its supplementary information files and from the corresponding author upon request.

## Code availability

The code and input files required to reproduce all the data in this study have been deposited in the GitHub repository LangevinDynamicsForPCP^51^.
