# Supplementary material for: Fluctuation-driven mass-selective transport in dynamic nanopores
Source: Nat Commun. 2026 Jul 15;17:8675. doi: 10.1038/s41467-026-75540-5 (PMC13490425; doi:10.1038/s41467-026-75540-5)
Supplement: Supplementary file 1 [file 41467_2026_75540_MOESM1_ESM.pdf]

Supplementary Information

Fluctuation-driven mass-selective transport in dynamic nanopores

Zhiye Tang,[1,2] Ken-ichi Otake,[3] Hirotoshi Sakamoto,[3] Susumu Kitagawa,[3,*] and Shinji Saito[1,2,*]

[1] Institute for Molecular Science, Myodaiji, Okazaki, Aichi 444-8585, Japan

[2] The Graduate University for Advanced Studies (SOKENDAI), Myodaiji, Okazaki, Aichi 444-8585, Japan

[3] Institute for Integrated Cell-Material Sciences, Institute for Advanced Study, Kyoto University (KUIAS-iCeMS), Kyoto 606-8501, Japan

Supplementary Notes 1 to 2
Supplementary Figures 1 to 5
Supplementary Tables 1 to 3
Supplementary Reference 1

[*] Corresponding authors: Susumu Kitagawa (kitagawa@icems.kyoto-u.ac.jp), Shinji Saito (shinji@ims.ac.jp)

Supplementary Note 1. Scaling law for predicting selectivity in high-barrier regimes

The energy barriers along the closed pathways in both [Cu(IDB)] and [Cu(OPTz)] are exceedingly high (Figs. 2i and 2j), making it computationally prohibitive to estimate isotopologue selectivities through direct numerical simulation of high-barrier systems. To overcome this, we constructed a series of model systems in which the barrier heights, V_C and V_O, were reduced while maintaining the original potential profile, i.e., a constant V_C/V_O ratio. The γ_η dependence of the selectivity D_1/D_2 for particles with masses $m_1 = 1$ and $m_2 = 2$ in the single-barrier [Cu(IDB)] model (Supplementary Fig. 1a) and double-barrier [Cu(IDB)] model (Supplementary Fig. 1b) is shown in Supplementary Figs. 2a and 2b, respectively.

The analysis of these low-barrier systems revealed a physically significant scaling law (Supplementary Figs. 2c–2f). Specifically, the peak position $\gamma_{\eta,max}(V_C)$, where D_1/D_2 reaches its maximum, exhibits a linear relationship with the barrier height V_C as $\gamma_{\eta,max}(V_C) = aV_C + b$. Furthermore, the peak height $(D_1/D_2)_{max}(V_C)$ follows a quadratic relationship, $(D_1/D_2)_{\max}(V_C) = c\, V_C^2 + d$, with high accuracy. The quadratic form is defined to satisfy $\left.\frac{\mathrm{d}(D_1/D_2)_{max}(V_C)}{\mathrm{d}V_C}\right|_{V_C=0} = 0$. The constant parameter d represents the limit at which the barrier vanishes, defined as $d = \sqrt{m_2/m_1}$ based on the mass dependence of Knudsen diffusion ($D \propto m^{-1/2}$). The coefficient of determination R^2 for both relations is nearly unity, suggesting that these scaling relations accurately describe the physical behavior of the peaks.

Importantly, the D_1/D_2 profiles obtained for different barrier heights (e.g., V_I and V_II) converged onto a single master curve when normalized by their respective peak positions and heights (Supplementary Figs. 2g and 2h),

$$\frac{\gamma_{\eta,V_\mathrm{I}}}{\gamma_{\eta V_\mathrm{I},max}} = \frac{\gamma_{\eta,V_\mathrm{II}}}{\gamma_{\eta V_\mathrm{II},max}}\ , \tag{S1}$$

$$\frac{(D_1/D_2)_{V_\mathrm{I}}(\gamma_{\eta,V_\mathrm{I}}) - \sqrt{m_2/m_1}}{(D_1/D_2)_{V_\mathrm{I},max} - \sqrt{m_2/m_1}} = \frac{(D_1/D_2)_{V_\mathrm{II}}(\gamma_{\eta,V_\mathrm{II}}) - \sqrt{m_2/m_1}}{(D_1/D_2)_{V_\mathrm{II},max} - \sqrt{m_2/m_1}}\,. \tag{S2}$$

Here, $\gamma_{\eta V_i,max}$ and $(D_1/D_2)_{V_i,max}$ denote the peak position and height of D_1/D_2, for a system with barrier height V_i, respectively, and $(D_1/D_2)_{V_i}(\gamma_\eta)$ represents the γ_η-dependent D_1/D_2 for that system. The existence of this master curve implies that the fundamental physical mechanism driving isotopologue separation remains invariant, regardless of the barrier height.

Based on these robust scaling relations, we estimated the γ_η dependence of the isotopologue selectivity for H_2O ($m_1 = 1$) and D_2O ($m_2 = 20/18$) in high-barrier systems where direct calculation is infeasible: the single-barrier [Cu(IDB)] (Fig. 4f), single-barrier [Cu(OPTz)] (blue curve in Fig. 5d), and double-barrier [Cu(IDB)] (red curve in Fig. 5d). We first analyzed the dependence of the isotopologue selectivity on γ_η in a series of low-barrier systems listed in Supplementary Table 2. For each system, we determined the peak position, $\gamma_{\eta,max}$, and the corresponding peak value, $(D_1/D_2)_{\max}$. As shown in Figs. 4c and 4d, the coefficients of determination R^2 are nearly unity for both $\gamma_{\eta,max}(V_C)$ and $(D_1/D_2)_{max}(V_C)$, confirming that both $\gamma_{\eta,max}$ and $(D_1/D_2)_{max}(V_C)$ accurately follow the scaling relations described above. Furthermore, the D_1/D_2 profiles for systems with different barrier heights (Supplementary Figs. 3a–3c) collapse onto a single master curve (Supplementary Figs. 3d–3f). These results demonstrate that the scaling laws and convergence to the master curve remain highly accurate even for systems with small mass ratios, such as $m_2/m_1 = 20/18$. These results also confirm the physical validity of our extrapolation method and the high reliability of the resulting estimates. Through this process, the γ_η dependence of the isotopologue selectivity for the original system with a high-energy barrier, which is computationally inaccessible, can be quantitatively estimated using the following equations:

$$\gamma_{\eta,V_{\mathrm{High}}} = \frac{\gamma_{\eta,V_{\mathrm{High}},max}}{\gamma_{\eta,V_{\mathrm{master}},max}} \gamma_{\eta,V_{\mathrm{master}}}, \tag{S3}$$

$$(D_1/D_2)_{V_{\mathrm{High}},max}\left(\gamma_{\eta,V_{\mathrm{High}}}\right) - \sqrt{m_2/m_1} = \frac{(D_1/D_2)_{V_{\mathrm{High}},max} - \sqrt{m_2/m_1}}{(D_1/D_2)_{V_{\mathrm{master}},max} - \sqrt{m_2/m_1}} \left\{(D_1/D_2)_{V_{\mathrm{master}}}(\gamma_{\eta,V_{\mathrm{master}}}) - \sqrt{m_2/m_1}\right\}. \tag{S4}$$

Supplementary Note 2. Scaling law for predicting self-diffusion coefficients in high-barrier regimes

As described in Supplementary Note 1, the high-energy barriers inherent to the target SPCs preclude direct evaluation of the self-diffusion coefficient D. To address this issue, we systematically scaled the barrier heights while preserving the relative potential landscape, i.e., a constant V_C/V_O ratio, as detailed in Supplementary Table 2, and estimated D for the original system via extrapolation. To ensure estimation accuracy in the high-barrier regime, where diffusivity is substantially reduced, we adopted an approach that incorporates analytical expressions whenever possible, rather than relying solely on numerical computations.

We consider a system in which the energy barrier fluctuates stochastically between the open and closed states at a rate γ_η. The self-diffusion of particles with masses m_1 and m_2 in this dynamic environment is described by $D_1(\gamma_\eta)$ and $D_2(\gamma_\eta)$. As detailed in the main text, $D_1(\gamma_\eta)$ and $\sqrt{m_2/m_1}D_2(\gamma_\eta)$ coincide in the fast-fluctuation limit and near the resonant activation (RA) regime (Supplementary Fig. 4a). In particular, near the RA regime, barrier-crossing dynamics are primarily governed by the lower V_O barrier. In this analysis, we calculated the self-diffusion coefficient $D_{1,o}$ under the potential V_O and the self-diffusion coefficient $D_{1,f}$ under the average potential (the mean of V_O and V_C) in the fast-fluctuation limit for a particle with mass m_1. Specifically, we analytically derived the mean first-passage time (MFPT), τ_{MFPT},[1] required to traverse a periodic length L of the system and calculated each self-diffusion coefficient using the relation $D = L^2/(2\tau_{\mathrm{MFPT}})$. Based on these results, we defined the logarithmic indicators $r_o = \log D_{1,o}$ and $r_f = \log D_{1,f}$.

Next, at the fluctuation rate $\gamma_{\eta,\mathrm{max}}$, where the selectivity D_1/D_2 reaches its maximum, we defined $r_1 = \log D_1(\gamma_{\eta,max})$ and $r_2 = \log(\sqrt{m_2/m_1}D_2(\gamma_{\eta,max}))$. We then introduced the average $r_{ave} = (r_1 + r_2)/2$ and deviation $\delta r = (r_1 - r_2)/2$, along with $\Delta r_+ = r_o - r_{ave}$ and $\Delta r_- = r_{ave} - r_f$. Finally, the difference $t = \Delta r_+ - \Delta r_-$ was fitted as a function of V_C. The parameter t quantifies the relative position of r_{ave} between

the analytical limits r_o and r_f, providing a stable metric for extrapolating D_1 in the high-energy barrier regime.

Supplementary Fig. 4b presents the V_C dependence of t determined from the self-diffusion of particles with masses of m_1 = 1 and m_2 =20/18 in the single-barrier [Cu(IDB)] system. The extrapolated t values for the original system, which has high-energy barriers, are also plotted. For extrapolation to the original high-energy system, three distinct functional forms were employed:

1. Generalized saturation exponential function: $t(V_C) = A[1 - \exp(-(kV_C)^p)]$
2. Hill function: $t(V_C) = AV_C^n/(B^n + V_C^n)$
3. Modified sigmoid function: $t(V_C) = A[a - \exp(-kV_C)]/[1 + a \exp(-kV_C)]$.

The coefficients of determination R^2 for all three functions are nearly unity, exceeding 0.999.

Once the parameter t is obtained, D_1 can be estimated accordingly. This extrapolation method for D_1 automatically also satisfies the D_1/D_2 ratio derived in Supplementary Note 1. Supplementary Table 3 summarizes the self-diffusion coefficient D_1 of H_2O at $\gamma_{\eta,max}$ for each model system, with $D_{1,f}$ and $D_{1,o}$ included as reference values. The value of D_1 for the single-barrier [Cu(IDB)] model is estimated to be 2.26–2.40×10^{-20} m^2 s^{-1}, depending on the extrapolation function. A comparison of D_1 calculated from these functions revealed that the discrepancy arising from the choice of functional form was at most approximately 6% (Supplementary Table 3). Furthermore, as shown in Supplementary Fig. 4c, the V_C dependence of D_1 is almost identical across these three functions. For the double-barrier [Cu(IDB)] model (corresponding to the red curve in Fig. 5d), the estimated D_1 values are 1.20–1.24×10^{-20} m^2 s^{-1}, whereas those for the [Cu(OPTz)] model (corresponding to the blue curve in Fig. 5d) are 1.18–1.20×10^{-15} m^2 s^{-1}. The variation in D_1 due to the fitting functions was less than 4% for the double-barrier [Cu(IDB)] and [Cu(OPTz)] models. These results confirm the robustness of the extrapolation method used in this study. The small D_1 values estimated for the SPCs, particularly in the [Cu(IDB)] models, reflect the very high-energy barriers for the water-diffusion process. Although the D_1 values remain very small in the high-barrier SPC models, dynamic barrier fluctuations

substantially enhance D_1 relative to the fast-fluctuation limit and drive selectivity far beyond the static mass-scaling limit.

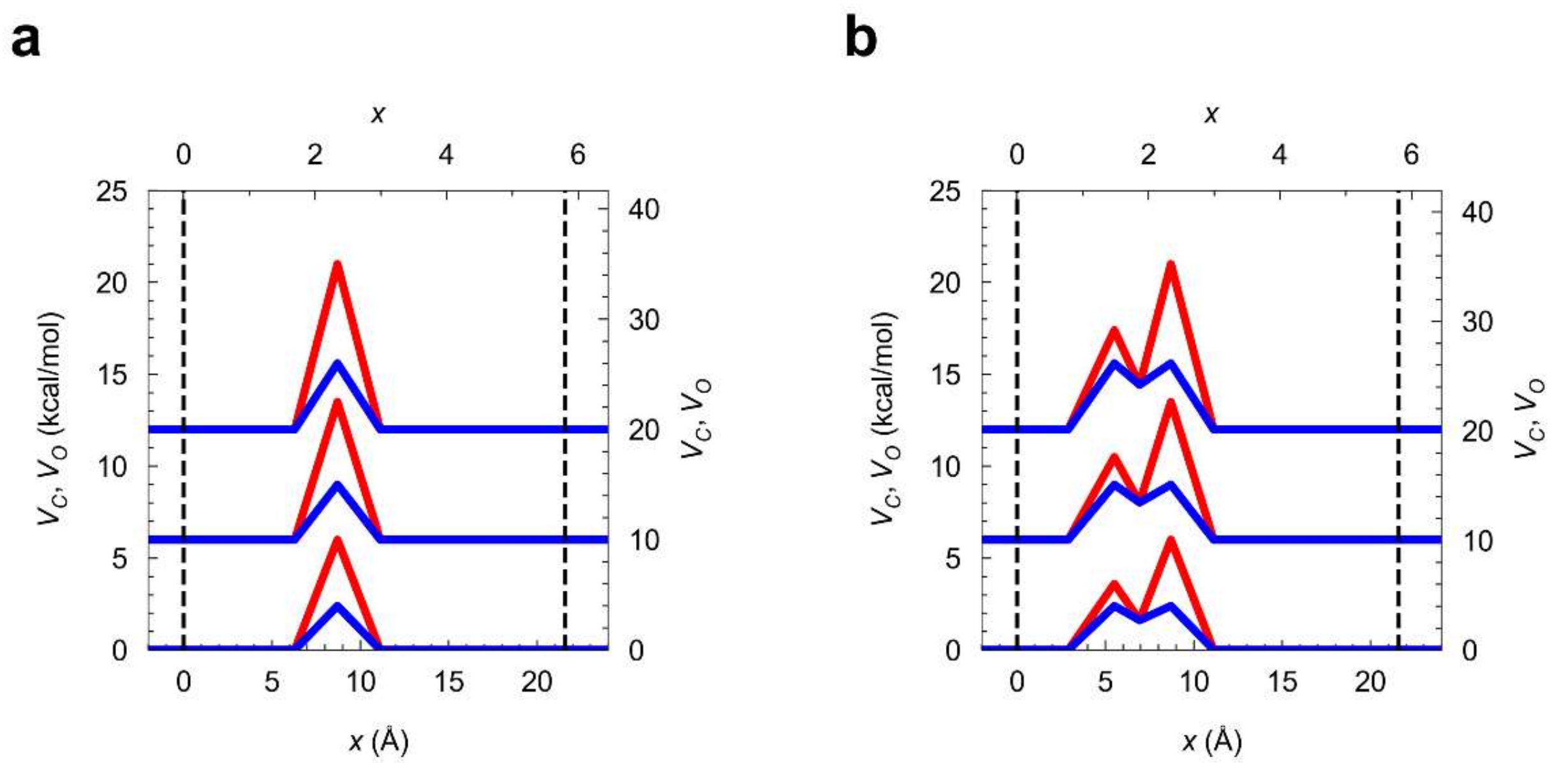

Supplementary Figure 1. Model potential energy profiles for the closed (red) and open (blue) pathways. **a**, Three single-barrier [Cu(IDB)] models with reduced barrier heights to facilitate computational sampling. These models, with a mass ratio of $m_2/m_1 = 2$, are used for the results shown in Figs. 3a, 3b, 3c, and 3d and Supplementary Figs. 2a, 2c, 2e, 2g. The barrier heights (V_C, V_O) are (6.0, 2.4), (7.5, 3.0), and (9.0, 3.6) kcal mol^{-1}. **b**, Three double-barrier [Cu(IDB)] models with reduced barrier heights. These models are used for Supplementary Figs. 2b, 2d, 2f, 2h. The barrier peaks are located at 5.5 Å (V_C = 3.6, V_O = 2.4 kcal mol^{-1}) and 8.7 Å (V_C = 6.0, V_O = 2.4 kcal mol^{-1}). For clarity, energy profiles in **a** and **b** are vertically offset. In all panels, vertical dashed lines indicate the boundaries of the periodic potentials. The periodic length, barrier positions, and widths are presented in Supplementary Table 1.

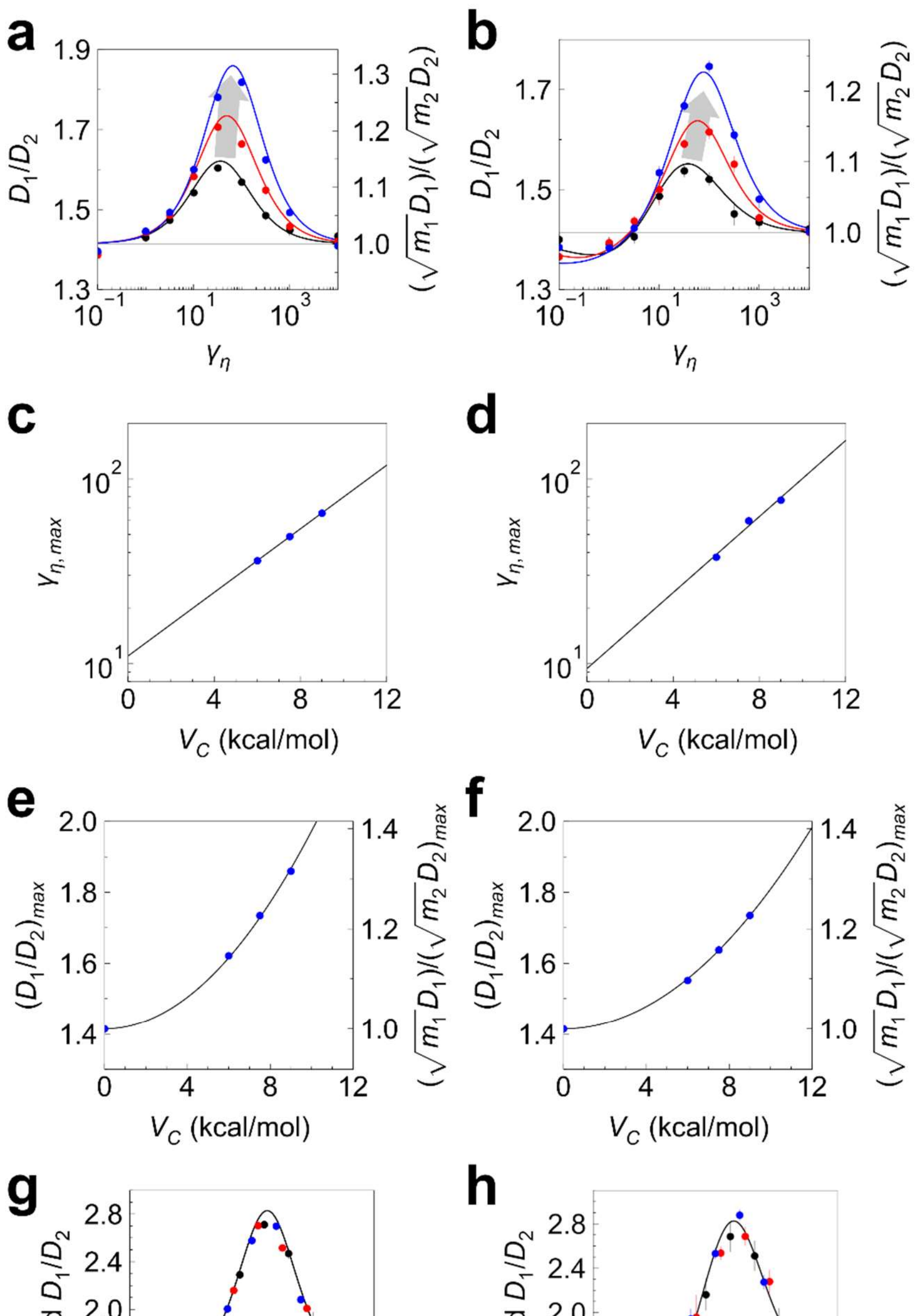

Supplementary Figure 2. Scaling behavior and extrapolation of isotopologue selectivity for particles with masses m_1 = 1 and m_2 = 2 in the single- and double-barrier [Cu(IDB)] models. **a**, **b**, γ_η dependence of selectivity in three low-barrier systems shown in Supplementary Figs. 1a (**a**) and 1b (**b**), respectively. Colored curves represent fits to the data for each system. Panel **a** is reproduced from Fig. 3d. **c**, **d**, Peak positions $\gamma_{\eta,max}$ as a function of V_C, corresponding to panels **a** and **b**, respectively. **e**, **f**, Peak heights $(D_1/D_2)_{max}$ as a function of V_C, corresponding to panels **a** and **b**, respectively. **g**, **h**, γ_η dependence of scaled selectivity, corresponding to **a** and **b**, respectively. Black curves represent fits to the scaled data across the three systems. Standard deviations are also scaled. In **c**–**f**, blue dots indicate calculated values for the low-barrier systems, whereas red dots represent extrapolated values for the original high-barrier system. Black curves represent fits to the calculated data; coefficients of determination (R^2) for the fits are 1.000 (**c**), 0.976 (**d**), 0.999 (**e**), and 1.000 (**f**), respectively. In **a**, **b**, **g**, and **h**, error bars represent standard deviations estimated using the bootstrap method.

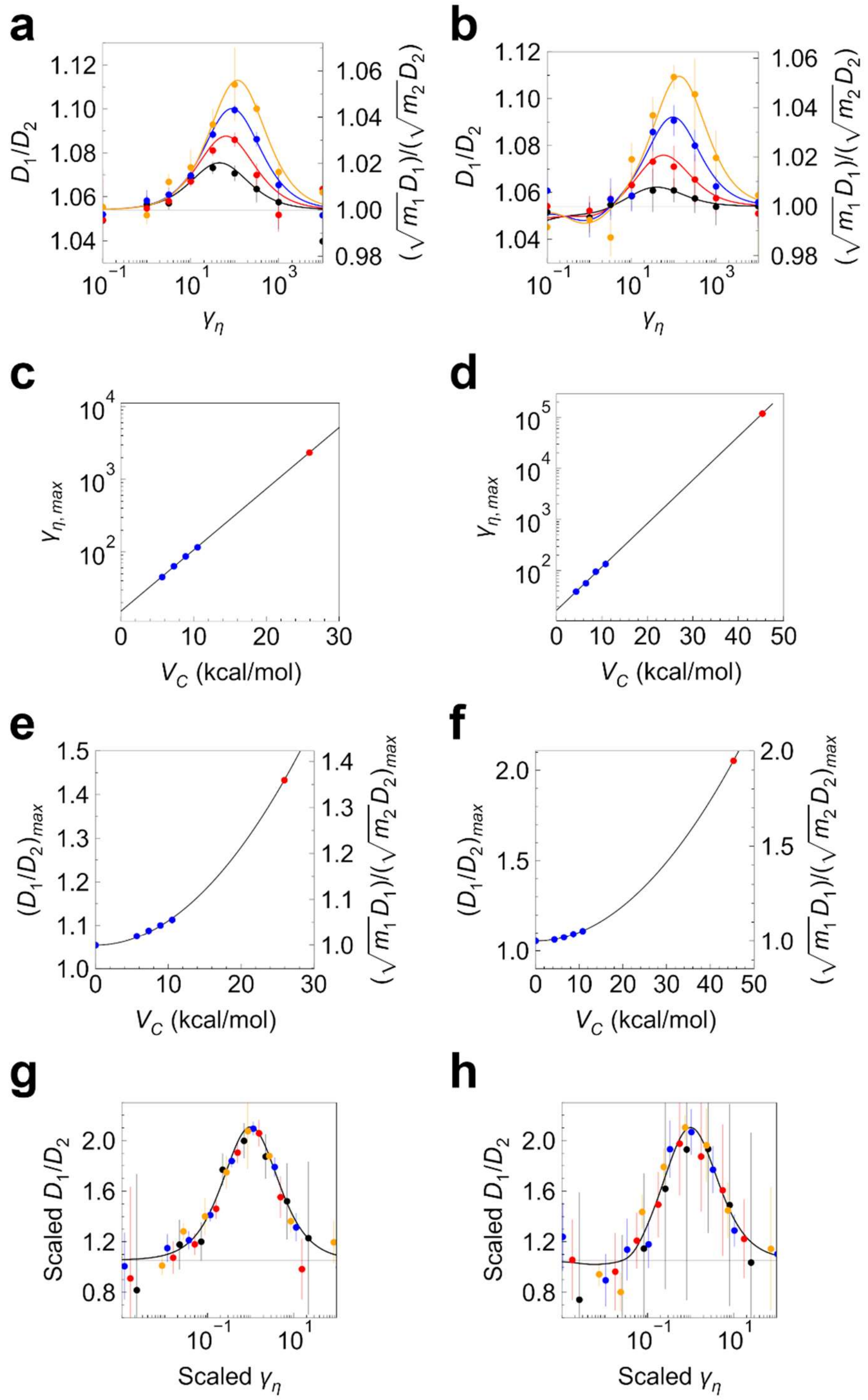

Supplementary Figure 3. Scaling behavior and extrapolation of isotopologue selectivity for particles with masses of m_1 = 1 and m_2 = 20/18 in the single-barrier [Cu(OPTz)] and double-barrier [Cu(IDB)] models. **a**, **b**, γ_η dependence of selectivity in four low-barrier systems listed in Supplementary Table 2. Colored curves represent fits to the data for each system. **c**, **d**, Peak positions $\gamma_{\eta,max}$ as a function of the closed-pathway barrier height V_C, corresponding to panels **a** and **b**, respectively. **e**, **f**, Peak heights $(D_1/D_2)_{max}$ as a function of V_C, corresponding to panels **a** and **b**, respectively. **g**, **h**, γ_η dependence of scaled selectivity, corresponding to panels **a** and **b**, respectively. Black curves represent fits to the scaled data across the four systems. Standard deviations are also scaled. In **c**–**f**, blue dots indicate calculated values for the low-barrier systems, whereas red dots represent extrapolated values for the original high-barrier system. Black curves represent fits to the calculated data; coefficients of determination (R^2) are 0.998 (**c**), 0.994 (**d**), 0.988 (**e**), and 0.996 (**f**). In **a**, **b**, **g**, and **h**, error bars represent standard deviations estimated using the bootstrap method.

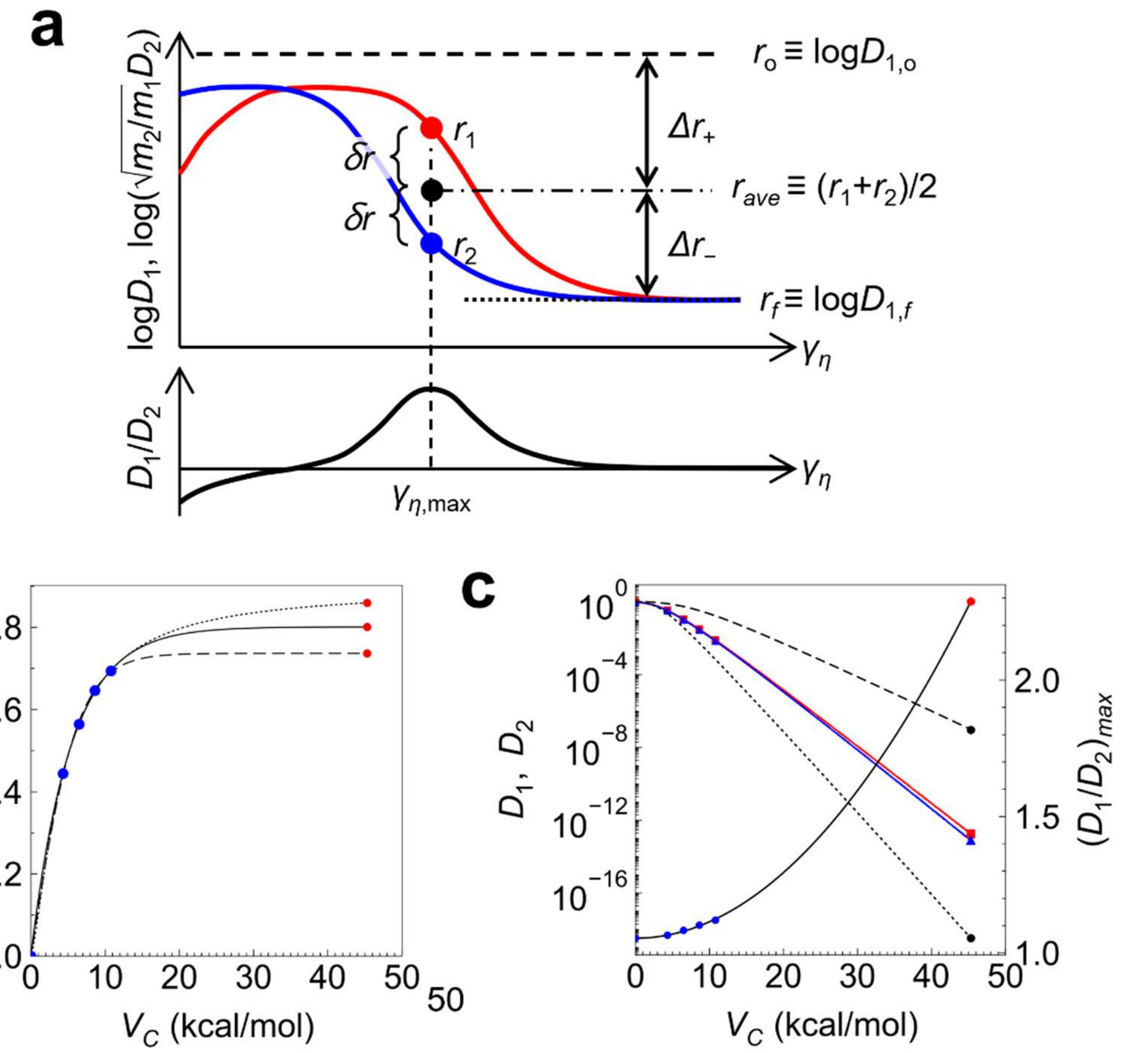

Supplementary Figure 4. Extrapolation scheme for self-diffusion coefficients in high-barrier regimes. **a**, Schematic of the scaling indicators used for extrapolation. At the fluctuation rate $\gamma_{\eta,\max}$, we define $r_1 = \log D_1(\gamma_{\eta,\max})$ and $r_2 = \log(\sqrt{m_2/m_1}D_2(\gamma_{\eta,\max}))$, and introduce $r_{ave} = (r_1 + r_2)/2$ and $\delta r = (r_1 - r_2)/2$. D_1 and D_2 denote self-diffusion coefficients for particles with masses m_1 and m_2, respectively. We further define $r_o = \log D_{1,o}$ for diffusion over the low barrier V_O, and $r_f = \log D_{1,f}$ for diffusion under the averaged potential in the fast-fluctuation limit, and quantify their relative positions by $\Delta r_+ = r_o - r_{ave}$ and $\Delta r_- = r_{ave} - r_f$. The extrapolation indicator is defined as $t = \Delta r_+ - \Delta r_-$. **b**, Dependence of t on V_C obtained from four scaled-down models of the single-barrier [Cu(IDB)] model (the first row in Supplementary Table 2), together with its extrapolation to the original high-barrier regime. Solid, dotted, and dashed curves represent fits to the four calculated data points using a generalized saturation exponential function, a Hill function, and a modified sigmoid function, respectively. All three functional forms give consistent extrapolated values, with coefficients of determination (R^2) greater than 0.999. **c**, V_C dependence of the extrapolated self-diffusion coefficients D_1 and D_2 for H_2O (m_1 = 1; red curve with circles) and D_2O (m_2 = 20/18; blue curve with triangles), together with the corresponding maximum D_1/D_2 values (right axis). Analytical self-diffusion coefficients for a particle of mass m_1 = 1 in the fast-fluctuation limit (dotted curve) and for diffusion over the low barrier V_O (dashed curve) are shown for comparison.

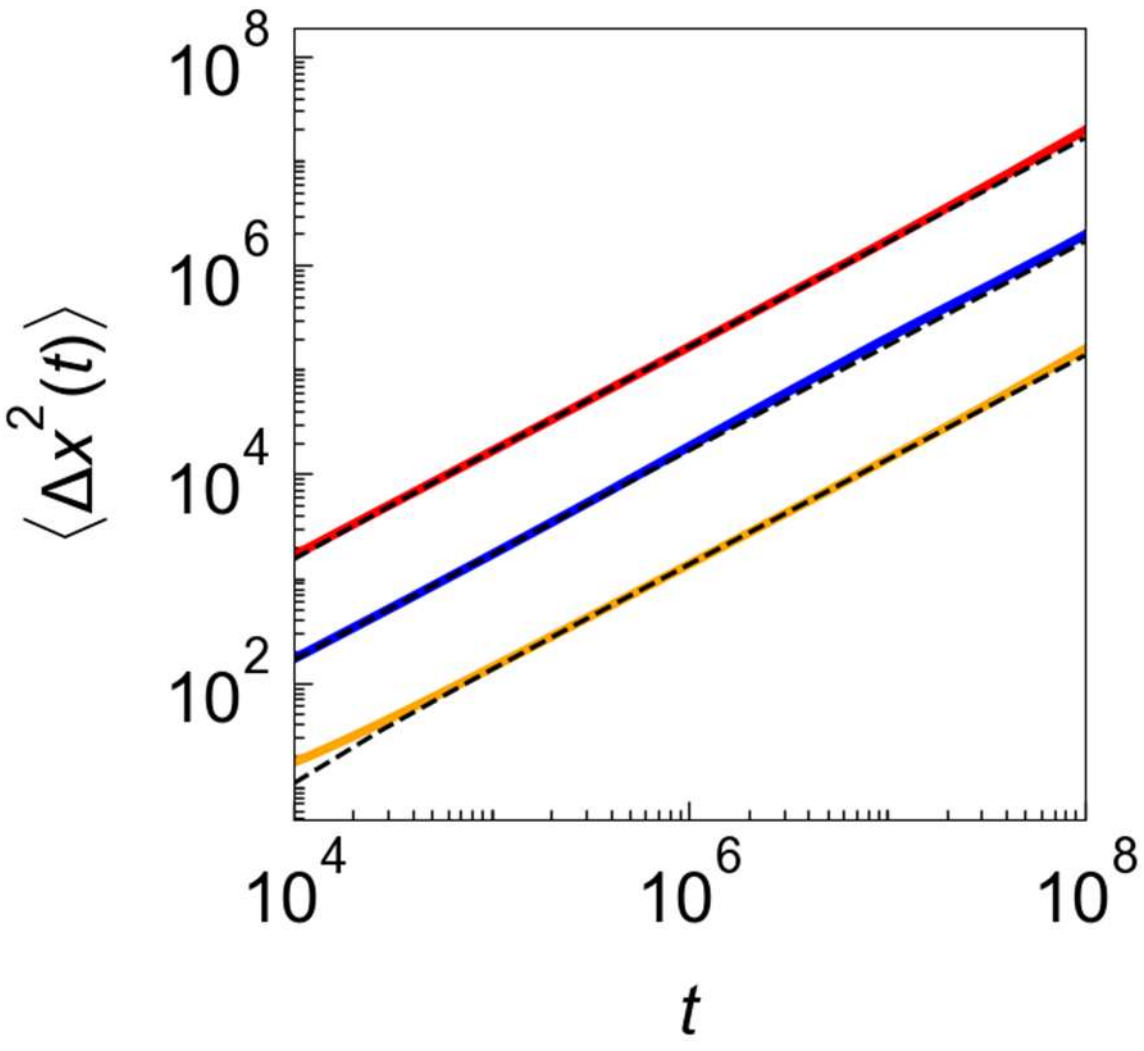

Supplementary Figure 5. Mean square displacement (MSD). MSD of a particle with mass 1 in the single-barrier [Cu(IDB)] model with barrier heights (V_C, V_O) = (10.73, 2.98) kcal mol^{-1}. Red, blue, and orange lines represent MSDs at γ_η = 10^{-1}, 10^1, and 10^5, respectively. Dashed black lines indicate linear fits, confirming the diffusive regime.

Supplementary Table 1. Model potential parameters for [Cu(IDB)] and [Cu(OPTz)].

	[Cu(IDB)]		[Cu(OPTz)]
Periodic length (Å)	21.7 (5.80)		7.8 (2.10)
Position of energy barrier (Å)	5.5 (1.48)	8.7 (2.34)	2.9 (0.78)
Width of energy barrier (Å)	5.2 (1.40)	4.8 (1.30)	4.5 (1.20)
Energy barrier height in closed pathway (kcal mol^{-1})	21.2 (35.5)	45.0 (75.5)	26.1 (43.8)
Energy barrier height in open pathway (kcal mol^{-1})	12.5 (21.0)	12.5 (21.0)	9.5 (16.0)

Values in parentheses denote scaled units (see Methods for details).

Supplementary Table 2. Energy barrier heights (V_C, V_O) for the four sets of scaled-down systems. All values are in kcal mol^{-1}.

Scaled-down systems for the target potential in Fig. 4a (Single-barrier [Cu(IDB)] model)			
(4.29, 1.19)	(6.44, 1.79)	(8.58, 2.38)	(10.73, 2.98)
Scaled-down systems for the target potential in Fig. 2i (First barrier of the double-barrier [Cu(IDB)] model)			
(2.03, 1.19)	(3.04, 1.79)	(4.05, 2.38)	(5.07, 2.98)
Scaled-down systems for the target potential in Fig. 2i (Second barrier of the double-barrier [Cu(IDB)] model)			
(4.29, 1.19)	(6.44, 1.79)	(8.58, 2.38)	(10.73, 2.98)
Scaled-down systems for the target potential in Fig. 2j (Single-barrier [Cu(OPTz)] model)			
(5.63, 2.09)	(7.24, 2.68)	(8.85, 3.28)	(10.46, 3.87)

The scaled-down systems were used to extrapolate the high-barrier target systems discussed in Fig. 4 and Supplementary Fig. 3. These low-barrier systems were specifically constructed to estimate the water isotopologue selectivity of the original high-barrier potentials.

Supplementary Table 3. Estimated self-diffusion coefficients of H_2O in the high-barrier models. Self-diffusion coefficients are reported in the scaled units used in Langevin-dynamics simulations. Values in parentheses are given in m^2 s^{-1}.

Model system	D_1[a]			$D_{1,f}$[e]	$D_{1,o}$[f]	D_1/D_2[g]
	Function 1[b]	Function 2[c]	Function 3[d]			
Single-barrier [Cu(IDB)] model (Fig. 4f)[h]	1.681×10^{-13} (2.326×10^{-20})	1.683×10^{-13} (2.258×10^{-20})	1.734×10^{-13} (2.401×10^{-20})	3.119×10^{-19} (4.316×10^{-26})	9.294×10^{-8} (1.286×10^{-14})	2.29
Double-barrier [Cu(IDB)] model (Red curve in Fig. 5d)[h]	8.779×10^{-14} (1.215×10^{-20})	8.644×10^{-14} (1.196×10^{-20})	8.972×10^{-14} (1.242×10^{-20})	3.533×10^{-19} (4.889×10^{-26})	2.435×10^{-8} (3.369×10^{-15})	2.05
[Cu(OPTz)] (Blue curve in Fig. 5d)[h]	8.619×10^{-9} (1.193×10^{-15})	8.550×10^{-9} (1.183×10^{-15})	8.636×10^{-9} (1.195×10^{-15})	1.615×10^{-11} (2.234×10^{-18})	6.787×10^{-6} (9.392×10^{-13})	1.43
Double-barrier [Cu(IDB)] model (Red curve in Fig. 5e)[i]	1.060×10^{-13} (1.467×10^{-20})	1.044×10^{-13} (1.445×10^{-20})	1.084×10^{-13} (1.500×10^{-20})	3.533×10^{-19} (4.889×10^{-26})	2.435×10^{-8} (3.369×10^{-15})	3.14
[Cu(OPTz)] model (Blue curve in Fig. 5e)[i]	9.367×10^{-9} (1.296×10^{-15})	9.121×10^{-9} (1.262×10^{-15})	9.411×10^{-9} (1.302×10^{-15})	1.615×10^{-11} (2.234×10^{-18})	6.787×10^{-6} (9.392×10^{-13})	1.88
Double-barrier [Cu(IDB)] model (Red curve in Fig. 5f)[j]	1.078×10^{-13} (1.492×10^{-20})	1.060×10^{-13} (1.467×10^{-20})	1.102×10^{-13} (1.524×10^{-20})	3.533×10^{-19} (4.889×10^{-26})	2.435×10^{-8} (3.369×10^{-15})	3.26
[Cu(OPTz)] model (Blue curve in Fig. 5f)[j]	9.438×10^{-9} (1.306×10^{-15})	9.181×10^{-9} (1.271×10^{-15})	9.486×10^{-9} (1.313×10^{-15})	1.615×10^{-11} (2.234×10^{-18})	6.787×10^{-6} (9.392×10^{-13})	1.93

[a]D_1 denotes the self-diffusion coefficient at the fluctuation rate $\gamma_{\eta,max}$ that maximizes isotopologue selectivity.
[b]Function 1 denotes a generalized saturation exponential function.
[c]Function 2 denotes a Hill function.
[d]Function 3 denotes a modified sigmoid function.
[e]$D_{1,f}$ denotes the self-diffusion coefficient in the fast-fluctuation limit.
[f]$D_{1,o}$ denotes the self-diffusion coefficient for diffusion on the open-pathway potential V_O.
[g]D_1/D_2 denotes the ratio of the self-diffusion coefficient of H_2O (m_1 = 1) to that of the heavier water isotopologue used in each system, namely D_2O (m_2 = 20/18) or T_2O (m_2 = 22/18).
[h]The heavier water isotopologue is D_2O, and the $m^{-1/2}$ scaling law is used for the self-diffusion coefficient.
[i]The heavier water isotopologue is T_2O, and the $m^{-1/2}$ scaling law is used for the self-diffusion coefficient.
[j]The heavier water isotopologue is D_2O, and the m^{-1} scaling law is used for the self-diffusion coefficient.

Supplementary Reference

1 Gardiner, C. W. *Handbook of Stochastic Methods*. Second edn, (Springer-Verlag, New York, 1985).
