# Supplementary material for: Fluctuation-driven mass-selective transport in dynamic nanopores
Source: Nat Commun. 2026 Jul 15;17:8675. doi: 10.1038/s41467-026-75540-5 (PMC13490425; doi:10.1038/s41467-026-75540-5)
Supplement: Supplementary file 2 [file 41467_2026_75540_MOESM2_ESM.pdf]

nature portfolio

# Peer Review File

# Fluctuation-driven mass-selective transport in dynamic nanopores

Corresponding Author: Professor Shinji Saito

**This file contains all reviewer reports in order by version, followed by all author rebuttals in order by version.**

Version 0:

Reviewer comments:

Reviewer #1

(Remarks to the Author)
The authors performed simplified dynamic simulations of diffusion in 1D potential models that were developed guided by quantum mechanical calculations. Using these methods, they compared the diffusion of water and heavy water in two different MOFs, one of which experimentally exhibits a dramatically different diffusion of the two host molecules and the other does not. They demonstrate general consistency with these experimental findings and ascribe the difference to dynamic fluctuations in a single barrier vs a double barrier pathway. This is an interesting topic and the theoretical approach appears to be sophisticated and appropriate. I felt that the methods and approaches were well described and could certainly provide a framework for understanding how framework fluctuations impact Knudsen diffusion.

A limitation in the manuscript is that there are many implicit and explicit simplifications and approximations employed by the authors in developing this framework, and it was unclear to me how success is judged. The graphical elements (figures) were underwhelming, and did not really help me understand the main findings, or the extent to which the theoretical observations were consistent (quantitatively) with previous experimental measurements. Also, because the entire method seemed aimed at explaining a single experimental observation, there is always the danger that the methods were optimized for this problem, and may not provide general predictive guidance. These criticisms do not negate the findings of the paper, but they may limit the impact of the work at this stage because the generality of the approach is currently unproven.

(Remarks on code availability)

Reviewer #2

(Remarks to the Author)
Referee Report on "Fluctuation-driven mass-selective transport in dynamic nanopores" by Tang et al., submitted to Nature Communications
An environmentally friendly gain of value-added products by separation is among the most profitable and beneficial applications of nanoporous materials. While, obviously, best conditions for their applicability are given for molecular mixtures with components notably differing in their characteristics including, in particular, their critical diameters, there are quite narrow limitations for operation with similarly shaped components. Within their present paper, the authors demonstrate that these narrow limitations may be overcome by a thoughtful exploitation of the pore dynamics, with particularly good options provided by the application of “soft porous crystals”, as notably represented by the metal-organic frameworks. Case studies, based on subtle differences in the masses of the components, unequivocally confirm this effect.
The paper is well written and refers to a topic of high relevance, deserving a broad audience as provided by Nature Communications. There are, however, a few items which should be cared for in a revision of this paper before publication. This refers, first, to the nomenclature. Throughout their paper, the authors use the term “diffusion coefficient”. What is meant, however, is in one case the coefficient of self-diffusion (or the self-diffusivity) – namely on considering the mean-square displacement of the guest molecules as a function of time, and in the other one the coefficient of transport diffusion (or the transport diffusivity) – namely on considering selectivities via permeabilities.
Importantly, both quantities are known to coincide in the limit of sufficiently small concentrations (namely, when mutual interaction of the guest molecules is negligibly small in comparison with their interaction with the host system). Thus, the conclusions drawn in this study are valid without any restrictions in the range of sufficiently low guest concentrations. A

corresponding remark and – ideally – some estimates of the influence of increasing guest concentration would, correspondingly, be highly desirable. This is all the more true because, in the interest of high performances, one should be particularly interested in higher concentrations.
Following the reasoning about „Diffusion anomaly in nanopores as a rich field for theorists and a challenge for experimentalists“ by S. Brandani et al. in Nature Communications 15 (2024) 5721, the interested reader will be curious to get some insight into the options – or, ideally, into first results – of the experimental confirmation of the presented results. Notably in this context, disclosure of the absolute values of the diffusivities appearing in figs. 3 and 5 and supplementary fig. 6 would be welcomed by the readers.
With these points referred to and/or taken account of in a revision, the paper is warmly recommended for publication

(Remarks on code availability)

Reviewer #3

(Remarks to the Author)
This manuscript presents a highly original and well-executed study. The authors provide a clear and quantitative explanation of the experimentally observed isotopologue selectivity in diffusion across two soft porous crystals by combining quantum chemical calculations with overdamped Langevin dynamics. In particular, rather than limiting their analysis to simplified model potentials, the authors determine realistic diffusion pathways and corresponding potentials for water molecules in actual experimental systems, namely [Cu(IDB)] and [Cu(OPTz)]. This enables them to directly connect their theoretical framework to real nanopore systems and convincingly explain the contrasting isotopologue selectivity observed in these materials.

Furthermore, the study does not merely demonstrate enhanced rates and diffusion coefficients within an optimized theoretical model but also incorporates normal mode analysis to estimate fluctuation rates in the experimental systems. This provides a sufficiently detailed and physically meaningful interpretation of the experimental observations.

From a theoretical perspective, the work is also significant in that it extends the concept of Resonant Activation from a single-barrier potential to a periodically modulated potential landscape. This advancement allows not only the evaluation of barrier-crossing rates but also the calculation of long-time diffusion coefficients, thereby broadening the applicability of the framework.

Overall, I believe that this manuscript provides meaningful physical insights and is suitable for publication in this journal after minor revisions.

Minor issues
1. Additional clarification in some parts would improve readability and help the reader better understand the interpretations:
- In the discussion of Figure 3, the authors state that *“in the slow-fluctuation limit, D1 converges to the weighted average of the diffusion coefficients of the individual pathways.”* It would be helpful to provide a brief explanation or derivation of why this relationship holds.

- In the interpretation of Figure 3, the authors state that the peak *“signifies accelerated transport assisted by energy fluctuations, when their timescale matches the transitionpath time of the low-barrier pathway.”* While the manuscript later discusses transitionpath time (or characteristic crossing time), no such information is available at this point
in the text. Therefore, it may be preferable to remove or defer this reference, depending on the authors’ judgment.

- The statement *“Widening the energy gap between $V_C(x)$ and $V_O(x)$ increases the probability of particle transition to the open pathway during barrier crossing, shifting the selectivity peak toward higher $\gamma_\eta$ values”* would benefit from further clarification or revision for improved clarity.

2. The authors determine transition pathways using the climbing-image nudged elastic band (CI-NEB) method. It would be beneficial to include a brief explanation of this method in the main text, along with a more detailed description in the Methods section or Supplementary Information.

3. Some figure references appear to be inconsistent:
- In the Supplementary Information, the single-barrier [Cu(OPTz)] (blue curve in Fig.

4d) and double-barrier [Cu(IDB)] (red curve in Fig. 4d) are mentioned, but these
correspond to Fig. 5d.

- In Supplementary Fig. 3, the values of $V_C$ and $V_O$ for the three low-barrier systems used in the single-barrier model of [Cu(IDB)] do not match those listed for the four low-barrier systems in Supplementary Table 2.

- Supplementary Table 2 refers to Supplementary Fig. 3b and Fig. 2g, and it is unclear whether these references are correct.

(Remarks on code availability)

Version 1:

Reviewer comments:

Reviewer #1

(Remarks to the Author)
The authors have clearly taken the comments of all reviewers to heart and through deeply about how the manuscript can be improved by addressed the reviewers' concerns, including my own. In particular, I feel that the revised manuscript does a much better job of explaining what it means for this method to be successful and how it may be extrapolated to other systems. I think it is suitable for publication in its current revised form.

(Remarks on code availability)

Reviewer #2

(Remarks to the Author)
In my understanding, the authors did a good job in referring to the reviewers' comments, both in responding to their concerns and in revising, accordingly, their paper. This is, in particular, true for their response to the points which I had mentioned in my review. I recommend acceptance as it stands and look forward to seeing the paper being published with Nature Communications.

(Remarks on code availability)

Reviewer #3

(Remarks to the Author)
I appreciate the authors' detailed responses and the thorough revision of the manuscript. The revised version is much clearer and easier to follow, and the results are now placed in an appropriate context. I warmly support publication of this revised work in Nature Communications.

(Remarks on code availability)

**Open Access** This Peer Review File is licensed under a Creative Commons Attribution 4.0 International License, which permits use, sharing, adaptation, distribution and reproduction in any medium or format, as long as you give appropriate credit to the original author(s) and the source, provide a link to the Creative Commons license, and indicate if changes were made.
In cases where reviewers are anonymous, credit should be given to 'Anonymous Referee' and the source.
The images or other third party material in this Peer Review File are included in the article’s Creative Commons license, unless indicated otherwise in a credit line to the material. If material is not included in the article’s Creative Commons license and your intended use is not permitted by statutory regulation or exceeds the permitted use, you will need to obtain permission directly from the copyright holder.
To view a copy of this license, visit https://creativecommons.org/licenses/by/4.0/

We sincerely thank all reviewers for their valuable comments, suggestions, and constructive criticism, which helped us improve the manuscript. We considered all the comments and suggestions carefully and revised our manuscript. Our point-by-point responses to the reviewers' comments are summarized below.

**Response to Reviewer #1**

**Comment 1:** The authors performed simplified dynamic simulations of diffusion in 1D potential models that were developed guided by quantum mechanical calculations. Using these methods, they compared the diffusion of water and heavy water in two different MOFs, one of which experimentally exhibits a dramatically different diffusion of the two host molecules and the other does not. They demonstrate general consistency with these experimental findings and ascribe the difference to dynamic fluctuations in a single barrier vs a double barrier pathway. This is an interesting topic and the theoretical approach appears to be sophisticated and appropriate. I felt that the methods and approaches were well described and could certainly provide a framework for understanding how framework fluctuations impact Knudsen diffusion.

**Response 1:** We thank Reviewer #1 for the positive assessment of our work and for recognizing that our theoretical approach provides a useful framework for understanding how framework fluctuations affect Knudsen diffusion in dynamic nanopores. We also appreciate the reviewer's constructive comments, which helped us clarify the scope of the model, improve the graphical presentation, and more clearly communicate the predictive and transferable aspects of the framework.

**Comment 2:** A limitation in the manuscript is that there are many implicit and explicit simplifications and approximations employed by the authors in developing this framework, and it was unclear to me how success is judged.

**Response 2:** We thank the reviewer for pointing out that the criteria for judging the success of the simplified framework were not sufficiently clear in the original manuscript. We agree that this point is important because the present model intentionally employs several simplifications: water diffusion in the SPC is described by an effective one-dimensional overdamped Langevin equation on a fluctuating energy landscape; the open and closed landscapes are represented by simplified piecewise-linear potentials derived from quantum-chemical calculations; and the model focuses on the dilute-loading, self-diffusion limit.

The purpose of these simplifications is not to reproduce all microscopic details of finite-loading transport, but to isolate the role of temporal energy-barrier fluctuations and determine whether this mechanism can account for mass-selective transport beyond the static Knudsen mass-scaling limit. We have now clarified this point in the revised manuscript.

We judge the success of the framework at three levels:

(i) whether the minimal model captures fluctuation-driven mass-selective transport on a fluctuating periodic landscape;

(ii) whether the SPC-specific calculations using independently computed quantum-chemical landscapes reproduce the contrasting isotope selectivities of [Cu(IDB)] and [Cu(OPTz)] without adjustable parameters; and

(iii) whether the analysis identifies transferable design principles for dynamic nanopores.

To make these criteria explicit, we revised the Results and Discussion section and strengthened the Conclusions to emphasize that the framework should be evaluated by whether it captures the dominant mechanism, explains the contrasting behavior of two SPCs, and provides general design principles for dynamic nanopores.

To clearly show the success of the present analysis, we have revised and added the following statements:

"*This result, obtained from quantum-chemical energy barriers with no adjustable parameters, is quantitatively consistent with the experimental selectivity (>2)*[31]*, demonstrating the predictive capability of our framework.*" (page 12, lines 3–6),

"*Together, these results demonstrate the predictive capability of the present framework. Using quantum-chemical energy landscapes without adjustable parameters, the model quantitatively reproduces the large $H_2O/D_2O$ selectivity observed in [Cu(IDB)] while explaining the weaker enhancement in [Cu(OPTz)]. Importantly, the analysis identifies two transferable design principles for dynamic nanopores: the magnitude of energy-barrier fluctuations between open and closed pathways and the alignment between the intrinsic nanopore dynamics and the energy-fluctuation rate that maximizes selectivity. These findings suggest that molecular transport properties can be rationally designed and controlled by actively manipulating system dynamics, either by tailoring intrinsic dynamics through ligand substitution or by applying external stimuli such as electric fields.*" (page 13, lines 21–29), and

"*By integrating our framework with the quantum-chemical energy landscapes of [Cu(IDB)] and [Cu(OPTz)], we provided a unified explanation for the contrasting isotopologue selectivities observed in these SPCs without introducing any adjustable parameters.*" (page 15, lines 4–6).

**Comment 3:** The graphical elements (figures) were underwhelming, and did not really help me understand the main findings, or the extent to which the theoretical observations were consistent (quantitatively) with previous experimental measurements.

**Response 3:** We thank the reviewer for this constructive comment. We agree that the original figures did not sufficiently clarify the main findings or the quantitative consistency with the previous experimental results. We have therefore substantially reorganized and improved the graphical presentation in the revised manuscript.

In **Fig. 1**, we added a new panel (Fig. 1b) showing diffusion over a static energy barrier, allowing direct comparison with diffusion over fluctuating barriers. This makes clear how dynamic barrier modulation amplifies the small mass difference between isotopologues beyond the static mass-scaling limit.

In **Fig. 2**, we moved the water diffusion pathways in [Cu(IDB)] and [Cu(OPTz)] from the Supplementary Information to the main text (Figs. 2a and 2b) and combined them with the gate structures and potential-energy profiles. This revision more clearly connects the quantum-chemical calculations and the experimentally relevant SPC structures to the simplified one-dimensional fluctuating potentials used in the model.

We also revised **Figs. 3 and 4** to improve the logical flow of the mechanistic explanation. Fig. 3 now focuses on the general resonant-activation mechanism, and Fig. 3a includes additional schematic illustrations of barrier crossing in the fast-fluctuation, slow-fluctuation, and resonant-activation regimes. The potential previously shown in Supplementary Fig. 3b has been moved to Fig. 4a, and the high-barrier single-barrier [Cu(IDB)] result previously shown in Fig. 3e has been moved to Fig. 4f. This arrangement places the high-barrier potential, the scaling analysis, and the extrapolated $H_2O/D_2O$ result in the same figure, thereby presenting the extrapolation procedure more coherently.

Most importantly, we revised **Fig. 5d** to explicitly show the quantitative comparison with experiment by adding an orange shaded region representing the $H_2O/D_2O$ selectivity estimated from the measurements by Su et al. The revised figure directly shows that the theoretical prediction for [Cu(IDB)] is consistent with the experimentally observed selectivity greater than 2, whereas [Cu(OPTz)] exhibits weaker enhancement.

We believe that the revised graphical structure makes the manuscript more self-contained, allowing readers to follow the logic from the conceptual mechanism, through the structural origin of the model potentials, to the quantitative comparison with experiment.

**Comment 4:** Also, because the entire method seemed aimed at explaining a single experimental observation, there is always the danger that the methods were optimized for this problem, and may not provide general predictive guidance. These criticisms do not negate the findings of the paper, but they may limit the impact of the work at this stage because the generality of the approach is currently unproven.

**Response 4:** We thank the reviewer for raising this important point. We agree that, because the model is simplified, it is essential to clarify whether the framework provides general predictive guidance rather than merely rationalizing a single experimental observation. In the revised manuscript, we have clarified that the SPC-specific calculations are not based on adjustable parameters fitted to the experimental selectivity. Instead, the energy landscapes used in the model are obtained independently from quantum-chemical calculations.

In the revised Results and Discussion section, we now explicitly state that the [Cu(IDB)] model reaches an $H_2O/D_2O$ selectivity of 2.05 using quantum-chemical energy barriers with no adjustable parameters, which is quantitatively consistent with the experimentally observed selectivity greater than 2:

"*This result, obtained from quantum-chemical energy barriers with no adjustable parameters, is quantitatively consistent with the experimental selectivity (>2)*[31]*, demonstrating the predictive capability of our framework.*" (page 12, lines 3–6).

Importantly, the same framework and protocol also explain the weaker enhancement in [Cu(OPTz)]. Thus, the model does not merely reproduce one positive observation, but captures the contrasting behavior of two SPCs using the same parameter-free procedure. To further address the reviewer's concern regarding general predictive guidance, we also clarified in the revised manuscript that the proposed mechanism is not restricted to a specific system or energy-landscape geometry. In the Conclusions, we have added the following sentence:

"*This analysis further established that the fluctuation-amplification mechanism persists across both single- and double-barrier landscapes and is therefore not restricted to a specific system or energy-landscape geometry.*" (page 15, lines 6–9).

We further emphasized that the framework provides transferable design principles rather than only a post hoc explanation of the [Cu(IDB)] experiment. Specifically, in the Results and Discussion section, we state that the framework identifies two design principles: the magnitude of energy-barrier fluctuations and the alignment between intrinsic nanopore dynamics and the energy-fluctuation rate that maximizes selectivity:

"*Importantly, the analysis identifies two transferable design principles for dynamic nanopores: the magnitude of energy-barrier fluctuations between open and closed pathways and the alignment between the intrinsic nanopore dynamics and the energy-fluctuation rate that maximizes selectivity*." (page 13, lines 24–27).

To further address the reviewer's concern that the framework may appear tailored to a single observation, we clarify here how the analyses presented in the manuscript support its generality.

First, before applying the framework to the SPC-specific systems, we established the mechanism in generic fluctuating periodic potentials and showed that resonant activation can enhance diffusion and amplify mass selectivity beyond the static mass-scaling limit.

Second, we showed that the same fluctuation-amplification mechanism persists in both single- and double-barrier landscapes, indicating that it is not restricted to a particular energy-landscape geometry.

Third, the extrapolation to high-barrier regimes is based on systematic scaling relations and master-curve collapse across multiple barrier heights, rather than empirical fitting to experimental selectivity. We now explicitly state this point in the Results and Discussion section:
"*This scaling behavior indicates that the underlying mechanism is invariant with respect to barrier height, providing evidence of generality*." (page 9, lines 10–11).

Fourth, we tested the robustness of the mechanism for $H_2O/T_2O$ and for an alternative mass-scaling assumption, confirming that the qualitative conclusion does not depend on a specific isotopologue pair or a particular mass-scaling law.

Fifth, we identified two transferable design principles for dynamic nanopores: the magnitude of energy-barrier fluctuations between open and closed pathways and the alignment between intrinsic nanopore dynamics and the energy-fluctuation rate that maximizes selectivity. These principles suggest that molecular transport properties in nanopores can be rationally designed and controlled by actively manipulating system dynamics, either through ligand substitution or external stimuli such as electric fields.

We believe that these revisions and clarifications address the reviewer's concern by making clear that the present framework is not a system-specific fit to a single observation, but rather a parameter-free and transferable framework that captures the dominant physical mechanism of fluctuation-driven mass selectivity.

**Response to Reviewer #2**

**Comment 1:** Referee Report on "Fluctuation-driven mass-selective transport in dynamic nanopores" by Tang et al., submitted to Nature Communications

An environmentally friendly gain of value-added products by separation is among the most profitable and beneficial applications of nanoporous materials. While, obviously, best conditions for their applicability are given for molecular mixtures with components notably differing in their characteristics including, in particular, their critical diameters, there are quite narrow limitations for operation with similarly shaped components. Within their present paper, the authors demonstrate that these narrow limitations may be overcome by a thoughtful exploitation of the pore dynamics, with particularly good options provided by the application of "soft porous crystals", as notably represented by the metal-organic frameworks. Case studies, based on subtle differences in the masses of the components, unequivocally confirm this effect.

The paper is well written and refers to a topic of high relevance, deserving a broad audience as provided by Nature Communications. There are, however, a few items which should be cared for in a revision of this paper before publication.

**Response 1:** We thank Reviewer #2 for the positive and encouraging assessment of our manuscript and for recognizing the broad relevance of our work. In the following responses, we have carefully addressed the items raised for revision, including the clarification of diffusion nomenclature, the consideration of concentration effects, and the disclosure of self-diffusion coefficients.

**Comment 2:** This refers, first, to the nomenclature. Throughout their paper, the authors use the term "diffusion coefficient". What is meant, however, is in one case the coefficient of self-diffusion (or the self-diffusivity) – namely on considering the mean-square displacement of the guest molecules as a function of time, and in the other one the coefficient of transport diffusion (or the transport diffusivity) – namely on considering selectivities via permeabilities.

Importantly, both quantities are known to coincide in the limit of sufficiently small concentrations (namely, when mutual interaction of the guest molecules is negligibly small in comparison with their interaction with the host system). Thus, the conclusions drawn in this study are valid without any restrictions in the range of sufficiently low guest concentrations. A corresponding remark and – ideally – some estimates of the influence of increasing guest concentration would, correspondingly, be highly desirable. This is all the more true because, in the interest of high performances, one should be particularly interested in higher concentrations.

**Response 2:** We thank the reviewer for pointing out the important distinction between self-diffusion and transport diffusion. In the revised manuscript, we now explicitly state that all diffusion coefficients calculated in the present one-dimensional Langevin simulations are self-diffusion coefficients, $D_s$. We also revised the terminology throughout the manuscript and Supplementary Information, replacing "diffusion coefficient" with "self-diffusion coefficient".

The present model describes the stochastic motion of a single particle on a fluctuating energy landscape. It does not explicitly include guest–guest interactions, excluded-volume effects, or loading-dependent changes in the effective potential. Therefore, the model corresponds to the dilute-loading limit. As noted by the reviewer, in this limit, $D_s$ coincides with the transport diffusion coefficient $D_t$, because guest–guest interactions are negligible. Accordingly, the present framework directly describes self-diffusion and provides the dilute-limit diffusivity component of transport selectivity.

We also added a statement on possible finite-loading effects. At finite loading, water–water interactions, collective transport effects, thermodynamic factors, and possible loading-dependent

changes in framework dynamics may modify $D_t$, even though the cavities of [Cu(IDB)] and [Cu(OPTz)] accommodate only a limited number of water molecules. We considered whether the influence of increasing guest concentration could be estimated within the same theoretical framework. However, because the present model describes single-particle motion on a one-dimensional fluctuating potential and does not explicitly represent molecular size, excluded-volume constraints, cavity occupancy, guest–guest interactions, or loading-dependent changes in the effective potential, adding multiple point particles to the same potential would not provide a physically meaningful estimate of finite-loading transport diffusion. We therefore refrained from presenting a quantitative finite-loading correction within this simplified model. Instead, we clarified the dilute-limit applicability of the framework and noted that quantitative evaluation of finite-loading effects will require future atomistic simulations that explicitly include guest–guest interactions and realistic pore geometry.

We have added the following explanation to the revised manuscript:
"*All diffusion coefficients calculated in the present framework are self-diffusion coefficients, $D_s$. This is because the model describes the stochastic motion of a single particle on a fluctuating energy landscape and does not explicitly include guest–guest interactions, excluded-volume effects, or loading-dependent changes in the effective potential. Thus, the framework corresponds to the dilute-loading limit, where guest–guest interactions are negligible and $D_s$ coincides with the transport diffusion coefficient $D_t$*[2,4,5]*. Accordingly, the present framework describes self-diffusion and provides the dilute-limit diffusivity component of transport selectivity. At finite loading, although the cavities of [Cu(IDB)] and [Cu(OPTz)] accommodate only a limited number of water molecules, estimated to be at most approximately five and six, respectively*[31]*, water–water interactions and associated collective and thermodynamic effects may modify $D_t$. Quantitative treatment of such effects will require future atomistic simulations that explicitly include guest–guest interactions and realistic pore geometry*." (page 5, line 26–page 6, line 4)

**Comment 3:** Following the reasoning about „Diffusion anomaly in nanopores as a rich field for theorists and a challenge for experimentalists" by S. Brandani et al. in Nature Communications 15 (2024) 5721, the interested reader will be curious to get some insight into the options – or, ideally, into first results – of the experimental confirmation of the presented results. Notably in this context, disclosure of the absolute values of the diffusivities appearing in figs. 3 and 5 and supplementary fig. 6 would be welcomed by the readers.
With these points referred to and/or taken account of in a revision, the paper is warmly recommended for publication.

**Response 3:** We thank the reviewer for this important and constructive suggestion. We agree that providing absolute self-diffusion coefficients is valuable for connecting the present theoretical results to future experimental validation, particularly in light of the recent discussion by Brandani et al. on diffusion anomalies in nanopores. In response to this comment, we have cited the work of Brandani et al. in the revised manuscript and explicitly connected our estimated self-diffusion coefficients to future experimental and computational tests of fluctuation-assisted transport in dynamic nanopores.

In the revised manuscript, we have estimated the self-diffusion coefficients for the high-barrier systems corresponding to Figs. 4f and 5d–5f. Because direct numerical evaluation of these coefficients is computationally prohibitive owing to the extremely high energy barriers in the target SPC models, we developed an extrapolation procedure based on systematically scaled low-barrier systems. This procedure is described in the newly added Supplementary Note 2 (pages 4–6), and the

extrapolation scheme is illustrated in Supplementary Fig. 4 (page 10). The estimated values are summarized in Supplementary Table 3 (page 14).

Specifically, for the single-barrier [Cu(IDB)] model shown in Fig. 4f, the $H_2O$ self-diffusion coefficient at the fluctuation rate that maximizes selectivity is estimated to be ~$2.3\times10^{-20}$ $m^2$ $s^{-1}$. For the systems shown in Fig. 5d, the corresponding values are ~$1.2\times10^{-20}$ $m^2$ $s^{-1}$ for [Cu(IDB)] and ~$1.2\times10^{-15}$ $m^2$ $s^{-1}$ for [Cu(OPTz)]. The corresponding values for Figs. 5e and 5f are also provided in Supplementary Table 3.

We have also added a discussion of the physical meaning of these values in the Results and Discussion section. The estimated self-diffusion coefficients are extremely small, particularly for [Cu(IDB)], reflecting the high energy barriers associated with water diffusion through the gated SPCs. Importantly, however, under such high-barrier conditions, dynamic barrier modulation can strongly amplify the subtle mass difference between $H_2O$ and $D_2O$, allowing the self-diffusion ratio to exceed the static mass-scaling limit. Thus, the small self-diffusivities and large selectivities are not contradictory; rather, they are two consequences of the same high-barrier, fluctuation-assisted transport mechanism.

Finally, following the reviewer's suggestion, we now explicitly state that these self-diffusion coefficients provide quantitative reference points for future experimental and computational tests of fluctuation-assisted transport in dynamic nanopores, consistent with the recent discussion on diffusion anomalies in nanopores.

We have added the following explanation to the revised manuscript:

"*We also estimated the self-diffusion coefficient of $H_2O$, $D_1$, at the fluctuation rate that maximizes selectivity for the high-barrier systems shown in Figs. 4f and 5d–5f. Because the energy barriers in these systems are extremely high, direct numerical evaluation of $D_1$ is computationally prohibitive; therefore, $D_1$ was evaluated using an extrapolation approach (Supplementary Note 2, Supplementary Fig. 4, and Supplementary Table 3). For the single-barrier [Cu(IDB)] model shown in Fig. 4f, $D_1$ is estimated to be ~$2.3\times10^{-20}$ $m^2$ $s^{-1}$. For the systems shown in Fig. 5d, $D_1$ is ~$1.2\times10^{-20}$ $m^2$ $s^{-1}$ for [Cu(IDB)] and ~$1.2\times10^{-15}$ $m^2$ $s^{-1}$ for [Cu(OPTz)]. The corresponding values for Figs. 5e and 5f are summarized in Supplementary Table 3. These extremely small self-diffusion coefficients, particularly for [Cu(IDB)], reflect the high-energy barriers associated with water diffusion through gated SPCs. Importantly, however, in this regime, dynamic barrier modulation can strongly amplify the subtle mass difference between $H_2O$ and $D_2O$, allowing the self-diffusion ratio to exceed the static mass-scaling limit. Thus, the small self-diffusivities and large selectivities are not contradictory; rather, they are two consequences of the same high-barrier, fluctuation-assisted transport mechanism. Consistent with the recent discussion on diffusion anomalies in nanopores[48], these self-diffusion coefficients provide quantitative reference points for future experimental and computational tests of fluctuation-assisted transport in dynamic nanopores.*" (page 13, lines 5–20)

**Response to Reviewer #3**

**Comment 1:** This manuscript presents a highly original and well-executed study. The authors provide a clear and quantitative explanation of the experimentally observed isotopologue selectivity in diffusion across two soft porous crystals by combining quantum chemical calculations with overdamped Langevin dynamics. In particular, rather than limiting their analysis to simplified model potentials, the authors determine realistic diffusion pathways and corresponding potentials for water molecules in actual experimental systems, namely [Cu(IDB)] and [Cu(OPTz)]. This enables them to directly connect their theoretical framework to real nanopore systems and convincingly explain the contrasting isotopologue selectivity observed in these materials.
Furthermore, the study does not merely demonstrate enhanced rates and diffusion coefficients within an optimized theoretical model but also incorporates normal mode analysis to estimate fluctuation rates in the experimental systems. This provides a sufficiently detailed and physically meaningful interpretation of the experimental observations.
From a theoretical perspective, the work is also significant in that it extends the concept of Resonant Activation from a single-barrier potential to a periodically modulated potential landscape. This advancement allows not only the evaluation of barrier-crossing rates but also the calculation of long-time diffusion coefficients, thereby broadening the applicability of the framework.
Overall, I believe that this manuscript provides meaningful physical insights and is suitable for publication in this journal after minor revisions.

**Response 1:** We are grateful to Reviewer #3 for the highly positive and encouraging assessment of our work. We also appreciate the reviewer's recognition of the originality of the study, the connection to experimental SPC systems, and the extension of resonant activation to long-time diffusion on periodically modulated landscapes. In the following responses, we have addressed the points raised by the reviewer.

**Comment 2:** Minor issues
1. Additional clarification in some parts would improve readability and help the reader better understand the interpretations:
- In the discussion of Figure 3, the authors state that "in the slow-fluctuation limit, D1 converges to the weighted average of the diffusion coefficients of the individual pathways." It would be helpful to provide a brief explanation or derivation of why this relationship holds.

**Response 2:** We thank the reviewer for this helpful comment. We agree that the explanation of the slow-fluctuation limit was insufficient, and we have revised the corresponding discussion in the main text.

In the slow-fluctuation limit ($\gamma_\eta \to 0$), the potential switching rate is significantly slower than the time required for diffusive traversal over one period. Therefore, a particle has sufficient time to diffuse within either the open or closed pathway before the potential switches. In this limit, the relevant quantity to average is not the diffusion coefficient itself, but the traversal time required to move over one periodic length $L$. If $\tau_o$ and $\tau_c$ denote the traversal times in the open and closed states, respectively, and the two states have equal occupancy probabilities, the average traversal time is $\tau_{\mathrm{avg}} = (\tau_o + \tau_c)/2$. Since $D_\alpha = L^2/(2\tau_\alpha)$ ($\alpha = o$ or $c$), this leads to $D_s^{-1} = (D_o^{-1}+D_c^{-1})/2$. Thus, the slow-fluctuation-limit value is given by the harmonic mean of the self-diffusion coefficients of the open and closed pathways, rather than their arithmetic mean.

We have corrected the text accordingly and added a brief explanation of this point in the discussion of Fig. 3:

"*In the slow-fluctuation limit ($\gamma_\eta \to 0$), the potential switching rate is much slower than the diffusive traversal of one period within the open and closed states. In this regime, the effective self-diffusion coefficient is determined by the average traversal time across one period. Because the open and closed states are equally populated in our model, $D_1$ can be expressed as the harmonic mean of the self-diffusion coefficients of the two pathways (black dotted line and slow-fluctuation limit panel in Fig. 3a).*" (page 7, lines 18–23).

**Comment 3:** - In the interpretation of Figure 3, the authors state that the peak "signifies accelerated transport assisted by energy fluctuations, when their timescale matches the transition path time of the low-barrier pathway." While the manuscript later discusses transition path time (or characteristic crossing time), no such information is available at this point in the text. Therefore, it may be preferable to remove or defer this reference, depending on the authors' judgment.

**Response 3:** We thank the reviewer for the helpful comment. We agree that introducing "transition path time" at this point could be confusing, as this concept is only introduced later.

We have revised the original sentence to remove "transition path time" while maintaining the physical interpretation of the peak as follows:
"*Crucially, $D_1(\gamma_\eta)$ does not vary monotonically between these limits; instead, it exhibits a pronounced maximum at approximately $\gamma_\eta \approx 0.4$, reflecting accelerated transport assisted by energy fluctuations, where barrier-crossing events are preferentially facilitated during transient low-barrier intervals*[38] *(resonant activation panel in Fig. 3a).*" (page 7, lines 23–27)

**Comment 4:** - The statement "Widening the energy gap between VC(x) and VO(x) increases the probability of particle transition to the open pathway during barrier crossing, shifting the selectivity peak toward higher γη values" would benefit from further clarification or revision for improved clarity.

**Response 4:** We thank the reviewer for this helpful comment. To clarify the physical mechanism, we have revised the relevant paragraph to explain how increasing the energy gap between $V_C(x)$ and $V_O(x)$ shifts the selectivity peak toward higher $\gamma_\eta$ values.

Specifically, we clarified that widening this energy gap increasingly suppresses barrier crossing in the closed state, so that successful crossing events become progressively confined to transient open-state intervals. Because lighter particles have shorter characteristic crossing times, they can more effectively exploit these shorter open-state windows than heavier particles. Consequently, as the energy gap widens, the optimal kinetic matching between the gate lifetime and the particle crossing time is achieved at higher $\gamma_\eta$, leading to a shift of the selectivity peak toward higher fluctuation rates.

We have revised the corresponding passage in the manuscript as follows:
"*Furthermore, increasing the energy gap between $V_C(x)$ and $V_O(x)$ shifts the selectivity peak $D_1/D_2$ toward higher $\gamma_\eta$ values. As the $V_C/V_O$ ratio increases, barrier crossing in the closed state is increasingly suppressed, and successful crossing becomes progressively restricted to transient intervals in the open state. While increasing $\gamma_\eta$ shortens the duration of each open-state interval, it increases the frequency with which particles encounter the open state*[43,44]*. Because a lighter particle has shorter characteristic crossing times, it can effectively exploit these shorter open-state intervals even at higher $\gamma_\eta$, whereas a heavier particle fails to complete its crossing before the barrier rises again. This mass-dependent matching between the gate lifetime and the particle crossing time shifts the selectivity peak toward higher $\gamma_\eta$ values as the energy gap widens.*" (page 8, lines 24–33)

**Comment 5:** 2. The authors determine transition pathways using the climbing-image nudged elastic band (CI-NEB) method. It would be beneficial to include a brief explanation of this method in the main text, along with a more detailed description in the Methods section or Supplementary Information.

**Response 5:** We thank the reviewer for this helpful suggestion.

In response, we have added a brief explanation of the CI-NEB method to the main text: "*This method identifies the minimum-energy path (MEP) by optimizing a series of intermediate configurations while driving the highest-energy image to the saddle point.*" (page 4, lines 19–20)

We have also added a more detailed description of the CI-NEB method in the Methods section: "*The CI-NEB method was used to determine the minimum-energy path (MEP) and transition state between the initial and final configurations. In this method, intermediate images are connected by virtual spring forces to maintain path continuity, whereas the nudged force projection prevents relaxation toward local minima. The climbing image scheme further refines the highest-energy image to locate the saddle point.*" (page 15, lines 25–29)

**Comment 6:** 3. Some figure references appear to be inconsistent:
- In the Supplementary Information, the single-barrier [Cu(OPTz)] (blue curve in Fig. 4d) and double-barrier [Cu(IDB)] (red curve in Fig. 4d) are mentioned, but these correspond to Fig. 5d.

**Response 6:** We thank the reviewer for pointing out this inconsistency. The reviewer is correct: the references to the single-barrier [Cu(OPTz)] model and the double-barrier [Cu(IDB)] model should have referred to Fig. 5d, not Fig. 4d.

We have corrected these figure references in the Supplementary Information as follows: "single-barrier [Cu(OPTz)] (blue curve in Fig. 5d), and double-barrier [Cu(IDB)] (red curve in Fig. 5d)" (page 3, lines 7–8 in Supplementary Information)

**Comment 7:** - In Supplementary Fig. 3, the values of VC and VO for the three low-barrier systems used in the single-barrier model of [Cu(IDB)] do not match those listed for the four low-barrier systems in Supplementary Table 2.

**Response 7:** We thank the reviewer for this helpful comment. We rechecked the $V_C$ and $V_O$ values and confirmed that there is no numerical inconsistency. The apparent mismatch arose because the three low-barrier systems shown in the original Supplementary Fig. 3 and the four low-barrier systems listed in Supplementary Table 2 were constructed for different analyses, but this distinction was not sufficiently explained in the main text and Supplementary Information.

In the revised Supplementary Information, the original Supplementary Fig. 3 has been reorganized as Supplementary Fig. 1 because the original Supplementary Figs. 1 and 2 have been moved to Figs. 2a and 2b.

We have clarified in the caption of Supplementary Fig. 1 that the three single-barrier models with $m_2/m_1 = 2$ are used for Figs. 3a–3d and Supplementary Figs. 2a, 2c, 2e, and 2g, whereas the corresponding double-barrier models are used for Supplementary Figs. 2b, 2d, 2f, and 2h.

We have also revised Supplementary Table 2 to state that the four scaled-down systems listed there were constructed separately to extrapolate the high-barrier water-isotopologue systems with $m_2/m_1 = 20/18$, as shown in Fig. 4 and Supplementary Fig. 3. In addition, we clarified the correspondence in the main text.

We have added the following explanation to the caption of Supplementary Fig. 1:

"*These models, with a mass ratio of $m_2/m_1 = 2$, are used for the results shown in Figs. 3a, 3b, 3c, and 3d and Supplementary Figs. 2a, 2c, 2e, 2g.*" and "*These models are used for Supplementary Figs. 2b, 2d, 2f, 2h.*" (page 7, lines 3–4 and line 6 in Supplementary Information).

We have also added the following explanation to the caption of Fig. 3:

"***c**, Self-diffusion coefficients $D_1$ (circles) and $\sqrt{2}D_2$ (squares) for three systems with ($V_C$, $V_O$) = (6.0, 2.4) (black), (7.5, 3.0) (red), and (9.0, 3.6) (blue) kcal mol$^{-1}$, shown in Supplementary Fig. 1a.*" (page 10, line 9 in Supplementary Information).

Regarding the explanation of Supplementary Table 2, we have added the following explanation to the footnote:

"*The scaled-down systems were used to extrapolate the high-barrier target systems discussed in Fig. 4 and Supplementary Fig. 3. These low-barrier systems were specifically constructed to estimate the water isotopologue selectivity of the original high-barrier potentials.*"

and revised the sentence describing the selectivity extrapolation in the single-barrier [Cu(IDB)] system as follows:

"*we estimated the selectivity by extrapolating results from four systems with systematically reduced barriers (see Supplementary Note 1 and Supplementary Table 2).*" (Page 9, line 4–6 in the main text).

We believe these revisions should clarify that the two sets of low-barrier models are distinct and serve different analyses.

**Comment 8:** - Supplementary Table 2 refers to Supplementary Fig. 3b and Fig. 2g, and it is unclear whether these references are correct.

**Response 8:** We thank the reviewer for this helpful comment. We rechecked the references and the $V_C$ and $V_O$ values and confirmed that there was no numerical inconsistency. However, we agree that the original presentation was difficult to follow.

In the revised manuscript, the original Supplementary Fig. 3 has been reorganized as Supplementary Fig. 1. The potential previously shown in Supplementary Fig. 3b has been moved to Fig. 4a, and the model potential previously referred to as Fig. 2g is now shown as Fig. 2i.

We have revised Supplementary Table 2 to clarify the correspondence between the scaled-down systems and the target potentials. Specifically, we have added a footnote explaining the systems listed in Supplementary Table 2:

"*The scaled-down systems were used to extrapolate the high-barrier target systems discussed in Fig. 4 and Supplementary Fig. 3. These low-barrier systems were specifically constructed to estimate the water isotopologue selectivity of the original high-barrier potentials.*" (page 13 in Supplementary Information)

We have also revised the row labels in Supplementary Table 2 so that each set of scaled-down systems is explicitly associated with the corresponding target potential. The revised row labels are:

"*Scaled-down systems for the target potential in Fig. 4a (Single-barrier [Cu(IDB)] model)*";

"*Scaled-down systems for the target potential in Fig. 2i (First barrier of the double-barrier [Cu(IDB)] model)*";

"*Scaled-down systems for the target potential in Fig. 2i (Second barrier of the double-barrier [Cu(IDB)] model)*"; and

"*Scaled-down systems for the target potential in Fig. 2j (Single-barrier [Cu(OPTz)] model)*" (page 13 in Supplementary Information).

We believe these revisions clarify the figure references and the correspondence between the scaled-down systems and the target models.
