# Supplementary material for: Fluctuation-driven mass-selective transport in dynamic nanopores
Source: Nat Commun. 2026 Jul 15;17:8675. doi: 10.1038/s41467-026-75540-5 (PMC13490425; doi:10.1038/s41467-026-75540-5)
Supplement: Supplementary file 3 [file 41467_2026_75540_MOESM3_ESM.pdf]

Description of Additional Supplementary Files

Supplementary Movie 1

Description: Structural changes at the second energy barrier for the closed (gray) and open pathways in [Cu(IDB)].

Supplementary Movie 2

Description: Structural changes at the energy barrier for the closed (gray) and open pathways in [Cu(OPTz)].

Supplementary Movie 3

Description: Vibrational normal mode associated with the structural change at the second energy barrier for the closed and open pathways in [Cu(IDB)]. The normal mode frequency is 17.9 cm^{-1}. Supplementary Movie 4. Vibrational normal mode associated with the structural change at the second energy barrier for the closed and open pathways in [Cu(IDB)]. The normal mode frequency is 36.8 cm^{-1}.

Supplementary Movie 5

Description: Vibrational normal mode associated with the structural change at the second energy barrier for the closed and open pathways in [Cu(IDB)]. The normal mode frequency is 38.3 cm^{-1}.

Supplementary Movie 6

Description: Vibrational normal mode associated with the structural change at the second energy barrier for the closed and open pathways in [Cu(IDB)]. The normal mode frequency is 40.2 cm^{-1}.

Supplementary Movie 7

Description: Vibrational normal mode associated with the structural change at the energy barrier for the closed and open pathways in [Cu(OPTz)]. The normal mode frequency is 12.5 cm^{-1}.

Supplementary Movie 8

Description: Vibrational normal mode associated with the structural transition at the energy barrier for the closed and open pathways in [Cu(OPTz)]. The normal mode

frequency is 17.7 cm−1.

Supplementary Movie 9

Description: Vibrational normal mode associated with the structural transition at the energy barrier for the closed and open pathways in [Cu(OPTz)]. The normal mode frequency is 19.3 cm−1.

Supplementary Movie 10

Description: Vibrational normal mode associated with the structural transition at the energy barrier for the closed and open pathways in [Cu(OPTz)]. The normal mode frequency is 40.1 cm−1.

Supplementary Movie 11

Description: Vibrational normal mode associated with the structural transition at the energy barrier for the closed and open pathways in [Cu(OPTz)]. The normal mode frequency is 42.1 cm−1.
